# Interventions to Minimize Unnecessary Antibiotic Use for Acute Otitis Media: A Meta-Analysis

**DOI:** 10.3390/children12101408

**Published:** 2025-10-17

**Authors:** Theresa L. Morin, Amy B. Stein, Rana E. El Feghaly, Amanda C. Nedved, Sophie E. Katz, Amy Keith, Heather E. Laferriere, Timothy C. Jenkins, Holly M. Frost

**Affiliations:** 1 Office of Research, Intermountain Health, Murray, UT 84107, USA; 2 Center for Health Systems Research, Denver Health and Hospital Authority, Denver, CO 80204, USA; 3 Department of Pediatrics, Children’s Mercy Kansas City, Kansas City, MO 64108, USA; relfeghaly@cmh.edu (R.E.E.F.); anedved@cmh.edu (A.C.N.); 4 Department of Pediatrics, University of Missouri-Kansas City, Kansas City, MO 64108, USA; 5 Department of Pediatrics, Vanderbilt University Medical Center, Nashville, TN 37204, USA; 6 Eskind Biomedical Library, Vanderbilt University, Nashville, TN 37204, USA; 7 Division of Infectious Diseases, Department of Medicine, Denver Health and Hospital Authority, Denver, CO 80204, USA; 8 Division of Infectious Diseases, Department of Medicine, University of Colorado School of Medicine, Aurora, CO 80045, USA; 9 Division of Infectious Diseases, Department of Pediatrics, University of Utah, Salt Lake City, UT 84132, USA

**Keywords:** acute otitis media, pediatrics, days of therapy

## Abstract

**Highlights:**

**What are the main findings?**

- Prescribing data for the treatment of acute otitis media (AOM) were abstracted from 83 studies in this meta-analysis.
- If prescribers followed the American Academy of Pediatrics guidelines for prescribing, annual antibiotic days of therapy (DOT) could be reduced by 60.6 million days (56%), while following the National Institutes for Health and Care Excellence guidelines for prescribing could reduce DOT by 76.7 million days (71%).

**What is the implication of the main finding?**

- Adherence to national guidelines for AOM management could avert millions of antibiotic DOT annually.
- Watchful waiting and short duration interventions have the greatest impact on antibiotic overprescribing.

**Abstract:**

**Backgrounds/Objectives:** Acute otitis media (AOM) is the leading reason antibiotics are prescribed to children. Despite guidelines advocating for watchful waiting and shorter antibiotic durations, overprescribing remains a concern. This meta-analysis aims to quantify potential reduction in antibiotic days of therapy (DOT) for AOM if prescribers adhered to guidelines. **Methods:** Cochrane databases were sourced for studies on ear infections, diagnostic accuracy, antibiotic duration, and watchful waiting. Randomized clinical trials, observational studies, and quality improvement reports of children aged 6 months–17 years with uncomplicated AOM published between 2000 and 2024 from the U.S., Canada, and Europe. Of 4187 studies, 425 met selection criteria. PRISMA guidelines were adhered to for independent extraction by multiple reviewers. Pooled prevalence of AOM outcomes and odds ratios (OR) for effectiveness interventions were calculated using the DerSimonian-Laird random effects model. A simulation study compared current practice to national guidelines. **Results:** Eighty-six studies found an estimated 107 million DOT prescribed to children in the U.S. annually for AOM. Following the American Academy of Pediatrics’ guidelines could reduce DOT by 57.9 million days (54%). Adherence to NICE guidelines could reduce DOT by 74.1 million days (70%). Watchful waiting and short-course antibiotic interventions had pooled Ors of 4.35 and 7.12, respectively, for decreasing DOT. **Conclusions**: Adherence to guidelines for AOM management could avert millions of antibiotic DOT. Watchful waiting and short-duration interventions are most impactful on antibiotic overprescribing.

## 1. Introduction

Acute otitis media (AOM) is the most frequent diagnosis associated with antibiotic prescribing in children [1]. Evidence-based guidelines available to healthcare providers in the United States and in Europe include recommendations for watchful waiting and shorter courses of antibiotic therapy as potential strategies for managing AOM [2,3]. Despite these guidelines, prescribers continue to overuse antibiotics with immediate prescriptions and longer courses than recommended. This overuse contributes to the broader public health issue of antibiotic resistance [4]. Further, antibiotic treatment is associated with adverse drug events. In addition, extraneous prescribing may result in widespread access concerns when antibiotic shortages occur [5]. The Centers for Disease Control and Prevention identified outpatient antibiotic stewardship programs as facilitators to reduce unnecessary antibiotic days of therapy (DOT) [6]. While stewardship programs have improved antibiotic prescribing, more research is needed to determine the type and focus of interventions that most effectively reduce antibiotic use in common pediatric infections, specifically AOM [7,8].

We conducted a systematic review and meta-analysis of current AOM prescribing behavior and treatment strategies. A simulation study was performed to identify the potential antibiotic DOT averted if prescribers adhered to national guidelines for the diagnosis and management of AOM. Additionally, we evaluated which antibiotic stewardship interventions had the greatest potential to reduce unnecessary antibiotic use for children with AOM.

## 2. Materials and Methods

### 2.1. Eligibility Criteria

This systematic review was limited to research performed in the United States, Canada, or Europe and published in English between 1 January 2000 and 30 June 2023. On 29 January 2025, we updated the search strategy to identify studies published between 30 June 2023 and 31 December 2024. Studies with data for AOM encounters or number of pediatric cases annually, proportions of diagnostic accuracy, disease classifications (e.g., severe, bilateral), and prescribing patterns such as watchful waiting, durations of antibiotic therapy, and the use of shared decision making were included. Additionally, we assessed studies reporting on the effectiveness of stewardship interventions for AOM, including those focused on diagnostic accuracy, antibiotic duration, use of watchful waiting strategies, and implementation of shared decision making. Studies of children aged between 6 months and 17 years (inclusive) with uncomplicated AOM were included. Although the American Academy of Pediatrics (AAP) guidelines for AOM limit inclusion criteria to children 6 months to 12 years, we chose to include children through age 17 years because overprescribing of antibiotics for AOM is prevalent in this population [9]. It is worth noting that the definition of severe AOM varied by study, but studies of uncomplicated AOM were included using the definition in the AAP guidelines [2]. We included randomized clinical trials, observational studies, and reports of quality improvement/quality assessment work.

Studies were excluded if they exclusively evaluated children with tympanostomy tubes, chronic otitis media, recurrent otitis media, and children with higher risks of complicated otitis media, including those with cochlear implants, trisomy 21, 22q11 deletion syndrome (DiGeorge syndrome), and other craniofacial abnormalities. Conference and meeting abstracts were also excluded.

### 2.2. Information Sources and Search Strategy

**Figure 1:**
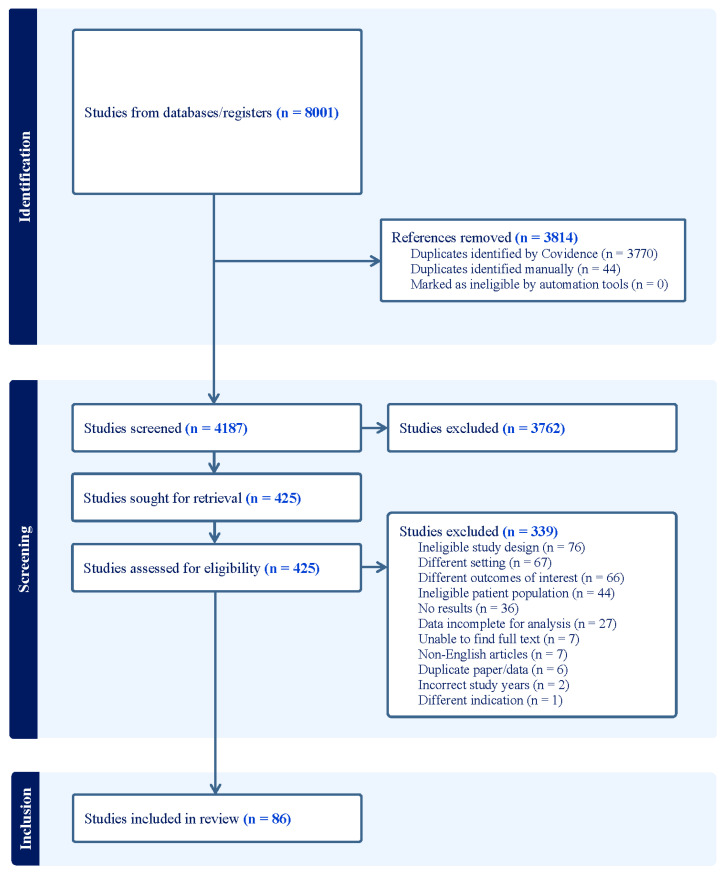
Study inclusion flow diagram.

Information sources included PubMed (NCBI), EMBASE (Elsevier), CINAHL Ultimate (EBSCOhost), Web of Science Core Collection (Clarivate), Cochrane Database of Systematic Reviews (Wiley), Cochrane CENTRAL (Wiley), reference lists of included papers and relevant systematic reviews. The original search was completed on 19 July 2023, (with an updated search performed on 29 January 2025), using a search strategy developed with assistance from a reference librarian (Supplementary Materials). A search strategy comprising a combination of keywords and database-specific subject headings included the following search terms and variations in each term: middle ear infection, middle ear inflammation, otitis media, antimicrobial stewardship, shared decision making, diagnostic errors, drug administration schedule, duration of therapy, guidelines, inappropriate prescribing, observation, unnecessary procedures, watchful waiting, amoxicillin, antibacterial agents, anti-infective agents, azithromycin, cefdinir, ceftriaxone, cefotaxime, ciprofloxacin, and penicillin. Articles were imported into Covidence, where 7 independent reviewers conducted article deduplication, abstract screening, and full-text review [10]. PRISMA guidelines were adhered to for independent extraction by multiple reviewers (Figure 1).

### 2.3. Data Collection and Bias Assessment

At least 2 reviewers completed abstract screening and full-text review for each article, and discordance was resolved by a third reviewer. Studies were critically appraised and evaluated for risk of bias and applicability using the CHARMS checklist [11]. Two reviewers independently collected relevant information in Covidence, including first author, publication year, article title, age distribution, number of pediatric AOM patients, and proportions and odds ratios (ORs) for outcomes of interest [10]. Following final article review, a biostatistician extracted data and performed qualitative and quantitative analysis.

### 2.4. Data Items

The primary objectives for this study were to (1) quantify the annual days of antibiotic use that could be averted if prescribers followed the 2013 AAP guidelines and/or the United Kingdom’s National Institute for Health and Care Excellence (NICE) guidelines for children (<18 years) with AOM, and to (2) identify which antibiotic stewardship approaches could have the largest impact on reducing antibiotic DOT for children with AOM. The United Kingdom’s guidelines were selected as a representative standard for many European countries [12]. Secondary objectives included annual days of antibiotic therapy prescribed for AOM in the United States, annual incidence of uncomplicated pediatric AOM in the United States, and potential days of antibiotics averted if prescribers accurately diagnosed AOM, used watchful waiting as the initial treatment approach, or prescribed a short duration of antibiotic therapy.

Variables of interest included study characteristics (study design, participant age ranges, exclusion criteria, and total number of participants), annual encounters for AOM in the United States (if available), diagnostic accuracy in the United States, duration of antibiotics prescribed in the United States (5 days, 7 days, and 10 days), use of watchful waiting for initial management of AOM (overall proportions, use of shared decision making, use of delayed prescriptions, and use of observation), proportion of severe cases of AOM, proportion of bilateral cases of AOM, proportion of children presenting for an AOM encounter by age range (<2 years, 2–5 years, >5 years), and intervention effectiveness data (diagnostic accuracy, duration of antibiotics prescribed, use of watchful waiting, and use of shared decision making). In articles without a clear definition of watchful waiting, the proportion was calculated by subtracting the reported prescribing rate from 100. If not reported, OR were calculated using counts in each group of interest.

We compared all prescribing data to AAP and NICE guidelines for AOM. A summary of these guidelines is provided in Table 1. When recommendations for either AAP or NICE guidelines included variable DOT (e.g., 5–7 days), the range of recommended durations (5 or 7 days) were factored into our analyses.

### 2.5. Statistical Analysis

Meta-analyses of the extracted data were analyzed to estimate the pooled prevalence of AOM outcomes and OR for effectiveness interventions. Associated 95% confidence intervals (CIs) and forest plots were calculated for all pooled estimates. The DerSimonian-Laird random effects model was used to generate the overall estimate. Heterogeneity measures were calculated using the I-squared index [13,14]. Sensitivity analyses were conducted by sequentially removing each study and reanalyzing the remaining data to evaluate the relative influence of each individual study on the pooled effect size. *p*-value of less than 0.05 was considered statistically significant and the meta package from R statistical software version 4.1.3 (R Foundation for Statistical Computing; Vienna, Austria) was used [15].

Using the estimates from the meta-analysis, we simulated days of antibiotic therapy based on the annual number of AOM encounters in the United States. The pooled prevalence estimates were combined based on the AAP and NICE guidelines. Reduction in DOT based on different interventions used the OR and assumed a base rate of 6% change in prescribing rate without any intervention [16]. The change based on the OR was applied to the DOT reduced by each component of the guidelines. Results are presented as DOT count, change, and percent reduction. The minimum and maximum DOT was obtained based on the range of annual encounters and the range of DOT recommended. Adherence to guidelines was considered using 6-day durations in order to find the average between guidelines for 5–7-day recommendations. The proportion of diagnostic accuracy, severity of infection, and bilateral infection was divided among annual encounters within each age group to determine the appropriate recommended DOT based on AAP or NICE guidelines.

## 3. Results

### 3.1. Meta-Analysis

A total of 8001 relevant studies were collected from databases and other sources based on the search strategies. We used Covidence and manual identification to remove 3814 duplicate studies [10]. Of the studies screened, we excluded 3762 based on the title and abstract and removed 339 after full-text review. Figure 1 contains the flow diagram for included and excluded studies and Table S1 details the data elements included from each study. In total, we identified 86 studies that included 1 or more usable data estimate for outcomes in this meta-analysis. Of these, 29 were cohort studies, 16 were randomized controlled trials, 11 were case series, and the remainder were surveys, retrospective, non-randomized trials, or quality improvement studies.

By meta-analysis, the pooled proportion of cases with an accurate diagnosis of AOM was 57% (95% CI 42.76, 70.43). The proportions of AOM cases prescribed 5-day, 7-day, and 10-day antibiotic durations were 2% (95% CI 0.00, 5.10), 11% (95% CI 1.69, 19.59), and 84% (95% CI 74.98, 93.24), respectively. Approximately 43% (95% CI 31.18, 54.81) of AOM encounters were severe and 34% (95% CI 23.42, 44.46) were bilateral. The proportions of AOM encounters for children <2 years old, ages 2–5 years, and age >5 years were 35% (95% CI 25.67, 44.61), 37% (95% CI 26.25, 47.76), and 25% (95% CI 20.40, 30.00), respectively (Table 2).

**Figure 2:**
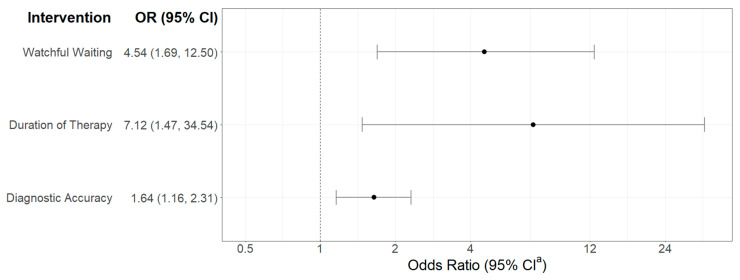
Odds ratio (OR) plot showing the pooled OR and 95% Confidence Interval (CI) for the effectiveness of 3 interventions to reduce the use and prescribing of antibiotics. Watchful waiting was considered in 10 studies, duration of therapy in 2, and diagnostic accuracy in 4 studies. ^a^ CI: Confidence Interval.

In the meta-analysis of studies of interventions aimed at reducing antibiotic prescriptions or durations of therapy, 10 studies employed interventions to increase the use of watchful waiting, 4 focused on improving diagnostic accuracy of AOM, and 2 focused on shortening durations of therapy. Results showed a significant reduction in the use of antibiotics in AOM from all 3 types of intervention, with the greatest treatment effect resulting from interventions to increase watchful waiting and decrease prescribed durations of antibiotics (Figure 2). Sensitivity analyses showed that no single study had substantial influence on the estimated effect size, as effect sizes remained stable for each outcome when removing individual studies.

### 3.2. Simulation of Days of Therapy

**Figure 3:**
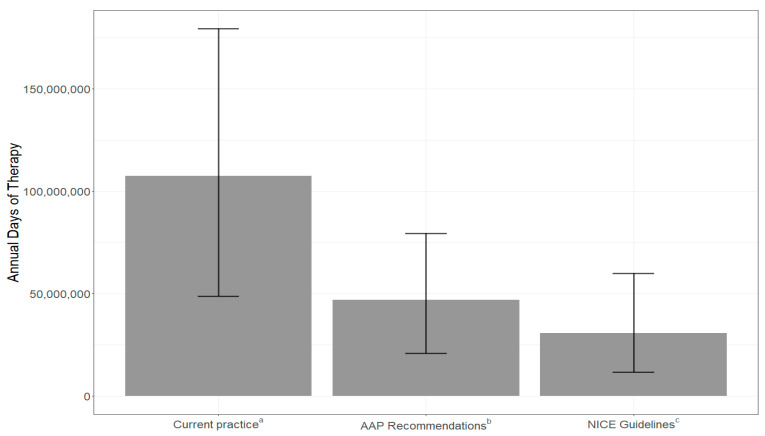
Total days of therapy based on 11.5 million annual AOM encounters and adherence to guidelines. The confidence intervals are calculated based on the range of AOM encounters (5.2 million and 19.2 million), and the range of days of therapy in AAP recommendations and NICE guidelines. ^a^ Current practice: estimated by pooling annual encounter data. ^b^ AAP: American Academy of Pediatrics. ^c^ NICE: National Institute for Health and Care Excellence.

Four studies between 2014 and 2021 estimated the annual number of encounters of AOM for the appropriate age range; the average was approximately 11.5 million with a range between 5.2 million and 19.2 million [17,18,19,20]. Based on our pooled estimates of the ages of children diagnosed with AOM, severity and laterality, and current antibiotic prescribing patterns (Table 3), an estimated 107 million days of antibiotic therapy are prescribed for AOM annually in the United States. If all AAP prescribing recommendations (Table 1) were followed, a 54% reduction in DOT would be achieved. If the NICE guidelines were followed, a 70% reduction in DOT would be achieved (Figure 3, Table S2).

Each aspect of the AAP and NICE guidelines were considered separately (Table 3). Accurate diagnosis resulted in a 43% decrease in DOT across recommendations. The adherence to the NICE guideline of use of watchful waiting (when antibiotics were prescribed) resulted in an approximate 46% decrease. Adherence to the AAP guidelines for correct duration of therapy and use of watchful waiting resulted in approximately 8% and 19% decreases in DOT, respectively. AAP guidelines recommend 10-day durations of therapy for all children aged 6–23 months and for severe infection in older age groups, thus we expect to see this impact in about 62% of the population. The results of this meta-analysis established current practice at 84% for 10-day prescriptions.

Finally, the pooled effectiveness of 3 types of interventions in comparison to current prescribing from the meta-analysis results were examined. The change associated with the OR was applied to the DOT reduced when considering the specific aspects of the AAP and NICE guidelines separately (Table 3). The highest averted days of antibiotic therapy were found with applying interventions to reduce durations of therapy (12.6 million days) and promote watchful waiting (10.7 million days) while following the NICE guidelines. Under the AAP recommendations, the interventions to reduce durations of therapy and promote watchful waiting resulted in smaller reductions (2.7 million and 4.5 million DOT). The final intervention type, aimed at increasing diagnostic accuracy, resulted in approximately 4.4 million averted DOT, as compared with current practice (Table 4).

## 4. Discussion

In this systematic review and meta-analysis, we estimated that 11.5 million visits for AOM in children occur each year, leading to approximately 107 million days of antibiotic therapy in the United States. Simulations demonstrate that if providers followed AAP guidelines for the diagnosis and management of AOM in children, days of antibiotic exposure could be reduced by more than half. Universal adherence to the NICE guidelines could result in nearly two-thirds of averted days of antibiotic exposure. All 3 intervention types analyzed in this study were associated with significant changes in the targeted prescribing behavior; however, the interventions promoting watchful waiting and reducing prescribed durations of antibiotic therapy were generally associated with a stronger treatment effect than interventions targeting diagnostic accuracy.

Our findings show that more than a decade since the last update to the AAP guidelines for AOM management, uptake for these recommendations is poor. The result is that children are exposed to twice the number of antibiotic therapy days necessary for treating AOM. Several factors are likely contributing to this problem, including difficulties in disseminating recommendations, caregiver and family desires to receive an antibiotic for ear infections, and a lack of scalable systems to facilitate implementation of guidelines into clinical practice. Integrating stewardship interventions into clinical practice poses challenges, commonly leading to longer patient visits and increased demand on multi-disciplinary resources, which are often limited in the healthcare industry. Interventions demonstrating success in reducing unnecessary prescribing of antibiotics need to be evaluated for scalability and adapted for real-time use in the clinical setting.

We found that inaccurate diagnosis of AOM contributed to a significant number of unnecessary antibiotic prescriptions for AOM, with the potential to avoid more than half of all exposure days. Despite this, interventions to improve diagnostic accuracy resulted in the lowest reduction in antibiotic DOT. Similarly to the trials analyzed in this study, previous research has indicated that interventions to improve diagnostic accuracy have not had sustainable impact [21]. Recently, digital otoscopy has been validated as a potential method to improve diagnostic accuracy in clinical trials, though the ability to reduce antibiotic use in clinical practice has not yet been demonstrated [21]. Access to validated and sustainable tools to improve diagnosis in real time is critical to improving antibiotic overuse in children with AOM.

In contrast, interventions focused on watchful waiting and antibiotic duration have been more successful in reducing antibiotic exposure days. Successful stewardship interventions for watchful waiting have included delayed antibiotic prescribing, prescriber education, clinical care pathways, decision support in the electronic health record, and audit and feedback to clinicians. These interventions have been evaluated using quasi-experimental and randomized controlled trials, with promising results [22,23,24,25,26]. Barriers to adoption of watchful waiting include caregiver pressure for a prescription and access to transportation to retrieve a prescription later if it is needed [27,28]. Interventions on antibiotic duration resulted in an even greater reduction in antibiotic DOT. These studies consistently used a multipronged approach, incorporating more than 1 stewardship mechanism to facilitate guideline adherence [22,29,30,31]. Additionally, the relative ease of changing duration fields in the electronic health record likely contributed to the success of these strategies. Interventions aimed at reducing duration also had a more sustainable impact, potentially due to both prescriber preference and parent satisfaction when treatment for AOM included some form of antibiotic prescription [32,33]. The main barriers to short-course therapy in primary care include a lack of awareness of prescribing guidelines and clinician concern that a short course may not be adequate to treat the present infection and prevent adverse outcomes (e.g., mastoiditis) and/or recurrence [34]. The strengths of this study include the ability to evaluate antibiotic use for AOM at a national level as well as a detailed examination of the effectiveness of antibiotic stewardship interventions. Additionally, the study design involved manual article review and consensus for data discordance among reviewers to achieve reliable data abstraction. Finally, for the simulation analysis, distinct components of each set of guidelines were considered separately to identify key focus areas for aligning prescribing behavior with recommendations.

This study also has some limitations. First, even with manual article review, the heterogeneity of studies for this population of interest could have skewed the proportion of individuals with true AOM. Second, the prevalence of appropriate antibiotic prescriptions was inconsistently reported and therefore not accounted for in the analysis. Third, reviewers were not blinded to authors or institutions of the studies assessed, which could have led to bias in the systematic review. Despite a large initial sample size, the inclusion criteria for this meta-analysis resulted in an insufficient number of studies to assess other potential stewardship interventions, such as shared decision making, that may warrant further research. Fourth, including only studies from the United States and European countries limits the generalizability of our findings to low-and middle-income regions. Fifth, it was not possible to establish an exact definition of severe AOM since the definition may have varied within each included study. Finally, we were not able to assess changes in antibiotic use or guideline concordance over time. However, Smolinski et al. found that the frequency of AOM diagnoses and the use of immediate antibiotics has increased, rather than decreased, since the publication of the 2013 AAP guidelines [16].

In conclusion, more than half of all prescribed antibiotic DOT for children with AOM in the United States could be avoided with adherence to national management guidelines. This finding highlights the urgent need to achieve meaningful changes in clinical practice for AOM. Interventions aimed at promoting watchful waiting and using short durations of therapy, in alignment with national guideline recommendations, are effective and need to be brought to scale. However, recent studies continue to highlight the difficulties with disseminating and implementing stewardship interventions to change antibiotic prescribing behavior [35,36,37,38]. Reduced days of antibiotic therapy due to alignment between guidelines and prescribing practice could also profoundly reduce cost [39]. A cost effectiveness assessment could be an important area for future study. AOM continues to be the most important target and greatest challenge in pediatric antimicrobial stewardship. We must foster ongoing collaborations between experts, frontline prescribers, and families to drive change.

## Figures and Tables

**Table 1:** AAP and NICE guidelines for prescribing by different presentations of acute otitis media (AOM) and patient age.

Presentation	Child Age	AAP ^a^ Guidelines	NICE ^b^ Guidelines
Unilateral, Non-Severe AOM	6–23 Months	10 Days of Antibiotics; Consider Watchful Waiting	Watchful Waiting; If Prescribing, Consider 5–7 Days of Antibiotics
Unilateral, Severe AOM	6–23 Months	10 Days of Antibiotics	5–7 Days of Antibiotics;
Bilateral, Severe and Non-Severe AOM	6–23 Months	10 Days of Antibiotics	5–7 Days of Antibiotics; Consider Watchful Waiting if Non-Severe
Unilateral and Bilateral, Non-Severe AOM	24+ Months	Consider Watchful Waiting; 7 Days of Antibiotics (children ages 2–5), 5–7 Days of Antibiotics (children ages 6+)	Watchful Waiting; If Prescribing, Consider 5–7 Days of Antibiotics
Unilateral and Bilateral, Severe AOM	24+ Months	10 Days of Antibiotics	5–7 days of Antibiotics

^a^ American Academy of Pediatrics. ^b^ National Institute for Health and Care Excellence.

**Table 2:** Meta-analysis of pooled estimate proportions of AOM outcomes among pediatric patients.

AOM Outcome	Number of Studies	Heterogeneity		Pooled Estimate; % (95% CI ^a^)
		I-Squared (%)	*p*-Value	
Diagnostic Accuracy	10	100%	<0.01	56.59 (42.76, 70.43)
Duration: 5 Days	3	99%	<0.01	2.29 (0.00, 5.10)
Duration: 7 Days	3	98%	<0.01	10.64 (1.69, 19.59
Duration: 10 Days	3	100%	<0.01	84.11 (74.98, 93.24)
Severe AOM ^b^	18	100%	<0.01	43.00 (31.18, 54.81)
Bilateral AOM ^b^	10	99%	<0.01	33.94 (23.42, 44.46)
AAP ^c^ Range: <2 years	18	100%	<0.01	35.14 (25.67, 44.61)
AAP ^c^ Range: 2–5 years	14	100%	<0.01	37.00 (26.25, 47.76)
AAP ^c^ Range: >5 years	13	100%	<0.01	25.20 (20.40, 30.00)
Use of Watchful Waiting	39	100%	<0.01	25.71 (18.44, 32.99)

^a^ CI: Confidence Interval. ^b^ AOM: Acute Otitis Media. ^c^ AAP: American Academy of Pediatrics.

**Table 3:** Estimated days of therapy (DOT) overall, saved, and percent decrease considering each recommendation of the guideline separately. The minimum DOT estimate is based on an annual AOM estimate of 5,200,000, and the maximum DOT estimate is based on an annual AOM estimate of 19,200,000. For the NICE and AAP guidelines, 5 days of therapy were used in the minimum calculation and 7 days of therapy in the maximum calculation (only when 5–7 days of therapy were the recommendation in the guideline).

		Days of Therapy	Minimum Days of Therapy	Maximum Days of Therapy
Current Practice		106,567,457	48,205,560	177,989,760
Correct Diagnosis		60,306,524	27,279,526	100,724,405
	Days of Therapy Saved	46,260,933	20,926,034	77,265,355
	% Decrease	43.4%	43.4%	43.4%
Correct Days of Therapy: AAP ^a^		98,019,085	43,592,120	166,471,296
	Days of Therapy Saved	8,547,652	4,613,440	11,518,464
	% Decrease	8.0%	9.6%	6.5%
Use of Watchful Waiting: AAP ^a^		86,032,749	38,361,836	145,741,373
	Days of Therapy Saved	20,534,708	9,843,724	32,248,387
	% Decrease	19.3%	20.4%	18.1%
Correct Days of Therapy: NICE ^b^		67,138,774	25,308,400	130,824,960
	Days of Therapy Saved	40,277,056	23,280,920	48,581,760
	% Decrease	37.5%	47.9%	27.1%
Use of Watchful Waiting: NICE ^b^		57,299,788	21,599,530	111,652,955
	Days of Therapy Saved	49,267,669	26,606,030	66,336,805
	% Decrease	46.2%	55.2%	37.3%

^a^ AAP: American Academy of Pediatrics. ^b^ NICE: National Institute for Health and Care Excellence.

**Table 4:** Table of the reduction in days of therapy (DOT) for 3 effectiveness interventions on current practice. These calculations assume a base rate of ~6% change in prescribing rate without intervention and are applied to the potential reduction for each aspect found in Table 3. The table gives the estimated DOT under current practice and the American Academy of Pediatrics (AAP) and National Institute for Health and Care Excellence (NICE) guidelines. The percent reduction represents the change from current practice estimate.

Intervention	Average DOT (Minimum–Maximum)	Reduction in DOT ^a^	% Reduction
Current Practice—No Intervention	106,567,457 (48,205,560–177,989,760)	Reference	Reference
Watchful Waiting: AAP ^b^	102,104,877 (46,066,333–170,959,844)	4,462,580	4.2%
Watchful Waiting: NICE ^c^	95,860,661 (42,423,567–163,573,518)	10,706,796	10.0%
Duration Therapy: AAP ^b^	103,896,628 (46,764,029–174,390,661)	2,670,829	2.5%
Duration Therap: NICE ^c^	93,982,350 (40,931,124–162,809,737)	12,585,107	11.8%
Diagnostic Accuracy	102,183,717 (46,222,585–170,668,004)	4,383,740	4.1%

^a^ The reduction in DOT is calculated from current practice with no intervention. ^b^ AAP: American Academy of Pediatrics. ^c^ NICE: National Institute for Health and Care Excellence.

## Data Availability

Deidentified individual participant data will not be made available.

## Supplementary Materials

The following supporting information can be downloaded at https://www.mdpi.com/article/10.3390/children12101408/s1: Figure S1: Preferred Reporting Items for Systematic Reviews and Meta-Analyses (PRISMA) Checklist; Figure S2: Preferred Reporting Items for Systematic Reviews and Meta-Analyses (PRISMA) Abstracts Checklist; Table S1: All included meta-analysis studies; Table S2: Overall total days of therapy saved from following AAP and NICE guidelines. References [40,41,42,43,44,45,46,47,48,49,50,51,52,53,54,55,56,57,58,59,60,61,62,63,64,65,66,67,68,69,70,71,72,73,74,75,76,77,78,79,80,81,82,83,84,85,86,87,88,89,90,91,92,93,94,95,96,97,98,99,100,101,102,103,104,105,106,107,108,109,110,111] are cited in the Supplementary Materials.

## Abbreviations

The following abbreviations are used in this manuscript:

AAP:	American Academy of Pediatrics
AOM:	Acute otitis media
CI:	Confidence interval
DOT:	Days of therapy
NICE:	National Institute for Health and Care Excellence
