# Supplementary material for: Interventions to Minimize Unnecessary Antibiotic Use for Acute Otitis Media: A Meta-Analysis
Source: Children (Basel). 2025 Oct 17;12(10):1408. doi: 10.3390/children12101408 (PMC12564321; doi:10.3390/children12101408)
Supplement: Supplementary file 1 [file children-12-01408-s001.zip › children-3832132-supplementary.pdf]

Supplement

Supplemental Methods. Search strategy for all databases sourced for the meta-analysis.

PubMed (NCBI): http://www.library.vanderbilt.edu/eres?id=145

Date Last Searched: 7/19/2023

Results: 1,846

Limits: Limited to children, Publication dates 1/1/2000-6/30/2023, Excluded conference/meeting abstracts

("Otitis Media"[Mesh:noexp] OR "Otitis Media, Suppurative"[Mesh] OR infected middle ear[tiab] OR infected middle ears[tiab] OR infectious middle ear[tiab] OR inflamed middle ear[tiab] OR inflammation of middle ear[tiab] OR inflammation of middle ears[tiab] OR inflammation of the middle ear[tiab] OR inflammation of the middle ears[tiab] OR inflammatory middle ear[tiab] OR inflammatory middle ears[tiab] OR media otitis[tiab] OR middle ear infect*[tiab] OR middle ear inflam*[tiab] OR middle otitis[tiab] OR otitis media*[tiab] OR otitis middle[tiab]) AND ("Antimicrobial Stewardship"[Mesh] OR "Decision Making, Shared"[Mesh] OR "Diagnostic Errors"[Mesh] OR "Drug Administration Schedule"[Mesh] OR "Duration of Therapy"[Mesh] OR "Guidelines as Topic"[Mesh] OR "Inappropriate Prescribing"[Mesh] OR "Observation"[Mesh] OR "Unnecessary Procedures"[Mesh] OR "Watchful Waiting"[Mesh] OR "1 course"[tiab] OR "2 courses"[tiab] OR "3 courses"[tiab] OR "4 courses"[tiab] OR "5 courses"[tiab] OR AAP recommend*[tiab] OR active surveillance[tiab] OR administration schedule[tiab] OR administration schedules[tiab] OR American Academy of Pediatrics[tiab] OR amoxicillin course[tiab] OR antibiotic course[tiab] OR antibiotic courses[tiab] OR antibiotic days[tiab] OR "antibiotics longer"[tiab] OR appropriate*[tiab] OR complete course[tiab] OR complete courses[tiab] OR complete drug[tiab] OR "complete drugs"[tiab] OR completed course[tiab] OR "completed drug"[tiab] OR

"completed oral*"[tiab] OR completed therap*[tiab] OR completed treatment[tiab] OR "completing antibiotic"[tiab] OR completing therap*[tiab] OR completing treatment[tiab] OR course amoxycillin[tiab] OR course antibacterial[tiab] OR course antibiotic[tiab] OR course antibiotics[tiab] OR course antimicrobial[tiab] OR course antimicrobials[tiab] OR course daily[tiab] OR course length*[tiab] OR "course of amoxicillin"[tiab] OR "course of amoxycillin"[tiab] OR "course of antibacterial"[tiab] OR "course of antibiotic"[tiab] OR "course of antibiotics"[tiab] OR "course of antimicrobial"[tiab] OR "course of antimicrobials"[tiab] OR "course of oral*"[tiab] OR "course of penicillin"[tiab] OR "course of therap*"[tiab] OR "course of treatment"[tiab] OR course oral*[tiab] OR course regimen[tiab] OR course regimens[tiab] OR "courses longer"[tiab] OR "courses of antibiotic"[tiab] OR "courses of antibiotics"[tiab] OR "courses of antimicrobial"[tiab] OR "courses of antimicrobials"[tiab] OR "courses of oral*"[tiab] OR "courses of therap*"[tiab] OR "courses of treatment"[tiab] OR courses prescribed[tiab] OR daily course[tiab] OR daily oral*[tiab] OR daily treatment[tiab] OR day amoxicillin[tiab] OR day amoxycillin[tiab] OR day antibiotic[tiab] OR day course[tiab] OR day oral*[tiab] OR day penicillin[tiab] OR day regimen[tiab] OR day regimens[tiab] OR day therap*[tiab] OR day treatment[tiab] OR days course[tiab] OR "days of amoxicillin"[tiab] OR "days of amoxycillin"[tiab] OR "days of antibiotic"[tiab] OR "days of antibiotics"[tiab] OR "days of oral*"[tiab] OR "days of therap*"[tiab] OR "days of treatment"[tiab] OR days therap*[tiab] OR days treatment[tiab] OR delay*[tiab] OR diagnoses criteria[tiab] OR diagnosing ability[tiab] OR diagnosing criteria[tiab] OR diagnosis abilities[tiab] OR diagnosis ability[tiab] OR diagnosis behavior*[tiab] OR diagnosis behaviour*[tiab] OR diagnosis criteria[tiab] OR diagnosis skill[tiab] OR diagnosis skills[tiab] OR diagnosis validity[tiab] OR diagnostic abilities[tiab] OR diagnostic ability[tiab] OR diagnostic behavior*[tiab] OR diagnostic behaviour*[tiab] OR diagnostic criteria[tiab] OR diagnostic criterion[tiab] OR diagnostic skill[tiab] OR diagnostic skills[tiab] OR diagnostic validity[tiab] OR diagnostical criteria[tiab] OR diagnostical criterion[tiab] OR diagnostical validity[tiab] OR "difficult to diagnose"[tiab] OR duration[tiab] OR durations[tiab] OR expectant[tiab] OR extended treatment[tiab] OR

extending treatment[tiab] OR failed diagnos*[tiab] OR "failure to diagnos*"[tiab] OR first course[tiab] OR first prescription[tiab] OR five courses[tiab] OR four courses[tiab] OR fourth course[tiab] OR full course[tiab] OR full courses[tiab] OR full treatment[tiab] OR full treatments[tiab] OR guidance[tiab] OR guideline[tiab] OR guidelines[tiab] OR immediacy[tiab] OR immediate[tiab] OR immediately[tiab] OR improper diagnos*[tiab] OR inappropriate*[tiab] OR initial amoxicillin[tiab] OR initial antibacterial[tiab] OR initial antibiotic[tiab] OR initial antibiotics[tiab] OR initial antimicrobial[tiab] OR initial course[tiab] OR initial drug[tiab] OR initial penicillin[tiab] OR initial therap*[tiab] OR initial treatment[tiab] OR initial treatments[tiab] OR judicious[tiab] OR "length of therap*"[tiab] OR "length of treatment"[tiab] OR long course[tiab] OR long courses[tiab] OR long drug[tiab] OR "long drugs"[tiab] OR long regimens[tiab] OR long term amoxicillin[tiab] OR long term antibiotic[tiab] OR long term antibiotics[tiab] OR long term penicillin[tiab] OR long term therap*[tiab] OR long term treatment[tiab] OR long term treatments[tiab] OR long treatment[tiab] OR longer course[tiab] OR longer courses[tiab] OR longer regimens[tiab] OR longer treatment[tiab] OR longer treatments[tiab] OR mis-diagnos*[tiab] OR misdiagnos*[tiab] OR missed diagnos*[tiab] OR month course[tiab] OR month treatment[tiab] OR monthly courses[tiab] OR "months of oral*"[tiab] OR "months of therap*"[tiab] OR "months of treatment"[tiab] OR months treatment[tiab] OR National Institute for Health and Care Excellence[tiab] OR national recommendation[tiab] OR national recommendations[tiab] OR NICE recommend*[tiab] OR nonguideline[tiab] OR "number of courses"[tiab] OR "number of prescribed"[tiab] OR "number of prescriptions"[tiab] OR observation[tiab] OR one course[tiab] OR one prescription[tiab] OR optimal length*[tiab] OR oral course[tiab] OR oral regimen[tiab] OR oral regimens[tiab] OR over-diagnos*[tiab] OR over-prescrib*[tiab] OR over-prescription[tiab] OR over-prescriptions[tiab] OR overdiagnos*[tiab] OR overprescrib*[tiab] OR overprescription[tiab] OR parent decision*[tiab] OR parents decision*[tiab] OR patient decision*[tiab] OR patients decision*[tiab] OR penicillin course[tiab] OR penicillin courses[tiab] OR prescribed daily[tiab] OR prescribing habits[tiab] OR prescribing practice[tiab] OR prescribing

practices[tiab] OR prescribing rate[tiab] OR prescribing rates[tiab] OR prescription habits[tiab] OR prescription length*[tiab] OR prescription practice[tiab] OR prescription practices[tiab] OR prescription rate[tiab] OR prescription rates[tiab] OR prescriptive practice[tiab] OR prescriptive practices[tiab] OR prolonged antibiotic[tiab] OR prolonged course[tiab] OR prolonged courses[tiab] OR prolonged medication[tiab] OR prolonged penicillin[tiab] OR prolonged therap*[tiab] OR prolonged treatment[tiab] OR proper diagnos*[tiab] OR properly diagnos*[tiab] OR prudent[tiab] OR "rate of antibiotic"[tiab] OR "rate of antibiotics"[tiab] OR "rate of prescribing"[tiab] OR "rate of prescription"[tiab] OR "rates of antibiotic"[tiab] OR "rates of antibiotics"[tiab] OR "rates of prescribing"[tiab] OR "regimens of antibiotic"[tiab] OR "regimens of antibiotics"[tiab] OR safety net antibiotic[tiab] OR second course[tiab] OR second prescription[tiab] OR second treatment[tiab] OR shared decision*[tiab] OR short antibiotic[tiab] OR "short antibiotics"[tiab] OR short course[tiab] OR short courses[tiab] OR short regimen[tiab] OR short regimens[tiab] OR short term antibiotic[tiab] OR short term antibiotics[tiab] OR short term oral*[tiab] OR short term penicillin[tiab] OR short term treatment[tiab] OR short term treatments[tiab] OR shortened antimicrobial[tiab] OR shortened course[tiab] OR shortened courses[tiab] OR shortened therap*[tiab] OR shortened treatment[tiab] OR shorter antibiotic[tiab] OR shorter course[tiab] OR shorter courses[tiab] OR shorter prescription[tiab] OR shorter regimen[tiab] OR shorter regimens[tiab] OR shorter treatment[tiab] OR standard courses[tiab] OR standard regimen[tiab] OR standard regimens[tiab] OR steward*[tiab] OR therapeutic recommendation[tiab] OR therapeutic recommendations[tiab] OR therapy courses[tiab] OR therapy recommendation[tiab] OR therapy regimens[tiab] OR therapy recommendations[tiab] OR three courses[tiab] OR treatment course[tiab] OR treatment courses[tiab] OR treatment day[tiab] OR treatment days[tiab] OR treatment length*[tiab] OR treatment recommendation[tiab] OR treatment recommendations[tiab] OR treatment regimen[tiab] OR treatment regimens[tiab] OR treatment time[tiab] OR two courses[tiab] OR two regimens[tiab] OR two treatments[tiab] OR under-diagnos*[tiab] OR under-prescrib*[tiab] OR under-prescription[tiab] OR

under-prescriptions[tiab] OR underdiagnos*[tiab] OR underprescrib*[tiab] OR underprescription[tiab] OR unnecessary[tiab] OR wait*[tiab] OR watchful[tiab] OR watching[tiab] OR week antibiotic[tiab] OR week course[tiab] OR weeks course[tiab] OR "weeks of antibiotic"[tiab] OR withheld[tiab] OR withhold*[tiab] OR (diagnos*[tiab] AND (accurac*[tiab] OR accurate*[tiab] OR blind spot[tiab] OR blind spots[tiab] OR capabilities[tiab] OR capability[tiab] OR certainties[tiab] OR certainty[tiab] OR correct[tiab] OR correctly[tiab] OR difficulties[tiab] OR difficulty[tiab] OR erroneous[tiab] OR error[tiab] OR errors[tiab] OR false[tiab] OR faulty[tiab] OR flawed[tiab] OR imprecise*[tiab] OR imprecision[tiab] OR inaccurac*[tiab] OR inaccurate*[tiab] OR incorrect[tiab] OR incorrectly[tiab] OR judgment[tiab] OR judgments[tiab] OR mistake*[tiab] OR precise*[tiab] OR precision[tiab] OR strict criteria[tiab] OR stringent criteria[tiab] OR uncertainties[tiab] OR uncertainty[tiab] OR wrong[tiab])) OR (("Drug Prescriptions"[Mesh] OR dispensing[tiab] OR prescribe*[tiab] OR prescribing[tiab] OR prescription[tiab] OR prescriptions[tiab] OR prescriptive[tiab]) AND ("Practice Patterns, Physicians'"[Mesh] OR annual[tiab] OR annually[tiab] OR behavior*[tiab] OR behaviour*[tiab] OR improper[tiab] OR improperly[tiab] OR over-consum*[tiab] OR over-use*[tiab] OR overconsum*[tiab] OR overuse*[tiab] OR pattern[tiab] OR patterns[tiab] OR proper[tiab] OR properly[tiab] OR trend[tiab] OR trends[tiab] OR under-consum*[tiab] OR under-use*[tiab] OR underconsum*[tiab] OR underuse*[tiab]))) AND ("Amoxicillin"[Mesh] OR "Anti-Bacterial Agents"[Mesh] OR "Anti-Infective Agents"[Mesh:noexp] OR "Azithromycin"[Mesh] OR "Cefdinir"[Mesh] OR "Ceftriaxone"[Mesh] OR "Cefotaxime"[Mesh] OR "Ciprofloxacin"[Mesh] OR "Penicillins"[Mesh] OR amoxicillin*[tiab] OR amoxicilline*[tiab] OR amoxy*[tiab] OR amoxycillin*[tiab] OR amoxycilline*[tiab] OR anti-bacteria*[tiab] OR anti-biotic*[tiab] OR anti-infect*[tiab] OR anti-microbial*[tiab] OR anti-mycobacteria*[tiab] OR antibacteria*[tiab] OR antibiotic*[tiab] OR antiinfect*[tiab] OR antimicrobial*[tiab] OR antimycobacteria*[tiab] OR azithromycin[tiab] OR azitromicina[tiab] OR azythromycin[tiab] OR bacteriocid*[tiab] OR cef triaxone[tiab] OR cefatriaxone[tiab] OR cefdinir[tiab] OR cefotaxim[tiab] OR cefotaxime[tiab] OR

cefotriaxone[tiab] OR ceftriaxon[tiab] OR ceftriaxone[tiab] OR cephotaxim[tiab] OR ciprofloxacin[tiab] OR microbicide[tiab] OR microbicides[tiab] OR penicillin[tiab] OR penicillins[tiab]) AND (2000/1/1:2023/6/30[pdat]) NOT ("Adult"[Mesh] NOT ("Adolescent"[Mesh] OR "Child"[Mesh] OR "Infant"[Mesh] OR "Pediatrics"[Mesh])) NOT "Meeting Abstract"[Publication Type]

Cumulative Index of Nursing and Allied Health Literature (CINAHL) Ultimate (EBSCOhost):

http://www.library.vanderbilt.edu/eres?id=29

Date Last Searched: 7/19/2023

Results: 668

Limits: Limited to children, Publication dates 1/1/2000-6/30/2023

(MH "Otitis Media" OR TI (infected middle ear OR infected middle ears OR infectious middle ear OR inflamed middle ear OR inflammation of middle ear OR inflammation of middle ears OR inflammation of the middle ear OR inflammation of the middle ears OR inflammatory middle ear OR inflammatory middle ears OR media otitis OR middle ear infect* OR middle ear inflam* OR middle otitis OR otitis media* OR otitis middle) OR AB (infected middle ear OR infected middle ears OR infectious middle ear OR inflamed middle ear OR inflammation of middle ear OR inflammation of middle ears OR inflammation of the middle ear OR inflammation of the middle ears OR inflammatory middle ear OR inflammatory middle ears OR media otitis OR middle ear infect* OR middle ear inflam* OR middle otitis OR otitis media* OR otitis middle)) AND (MH "Antimicrobial Stewardship" OR MH "Decision Making, Patient+" OR MH "Decision Making, Shared" OR MH "Diagnostic Errors+" OR MH "Drug Administration Schedule" OR MH "Inappropriate Prescribing" OR MH "Observational Methods+" OR MH "Practice Guidelines" OR MH "Prescribing Patterns" OR MH "Treatment Duration" OR MH "Unnecessary Procedures" OR TI ("1 course" OR "2 courses" OR "3 courses" OR "4 courses" OR "5 courses" OR AAP

recommend* OR active surveillance OR administration schedule OR administration schedules OR American Academy of Pediatrics OR amoxicillin course OR antibiotic course OR antibiotic courses OR antibiotic days OR "antibiotics longer" OR appropriate* OR complete course OR complete courses OR complete drug OR "complete drugs" OR completed course OR "completed drug" OR "completed oral*" OR completed therap* OR completed treatment OR "completing antibiotic" OR completing therap* OR completing treatment OR course amoxycillin OR course antibacterial OR course antibiotic OR course antibiotics OR course antimicrobial OR course antimicrobials OR course daily OR course length* OR "course of amoxicillin" OR "course of amoxycillin" OR "course of antibacterial" OR "course of antibiotic" OR "course of antibiotics" OR "course of antimicrobial" OR "course of antimicrobials" OR "course of oral*" OR "course of penicillin" OR "course of therap*" OR "course of treatment" OR course oral* OR course regimen OR course regimens OR "courses longer" OR "courses of antibiotic" OR "courses of antibiotics" OR "courses of antimicrobial" OR "courses of antimicrobials" OR "courses of oral*" OR "courses of therap*" OR "courses of treatment" OR courses prescribed OR daily course OR daily oral* OR daily treatment OR day amoxicillin OR day amoxycillin OR day antibiotic OR day course OR day oral* OR day penicillin OR day regimen OR day regimens OR day therap* OR day treatment OR days course OR "days of amoxicillin" OR "days of amoxycillin" OR "days of antibiotic" OR "days of antibiotics" OR "days of oral*" OR "days of therap*" OR "days of treatment" OR days therap* OR days treatment OR delay* OR diagnoses criteria OR diagnosing ability OR diagnosing criteria OR diagnosis abilities OR diagnosis ability OR diagnosis behavior* OR diagnosis behaviour* OR diagnosis criteria OR diagnosis skill OR diagnosis skills OR diagnosis validity OR diagnostic abilities OR diagnostic ability OR diagnostic behavior* OR diagnostic behaviour* OR diagnostic criteria OR diagnostic criterion OR diagnostic skill OR diagnostic skills OR diagnostic validity OR diagnostical criteria OR diagnostical criterion OR diagnostical validity OR "difficult to diagnose" OR duration OR durations OR expectant OR extended treatment OR extending treatment OR failed diagnos* OR "failure to diagnos*" OR first course OR first prescription OR

five courses OR four courses OR fourth course OR full course OR full courses OR full treatment OR full treatments OR guidance OR guideline OR guidelines OR immediacy OR immediate OR immediately OR improper diagnos* OR inappropriate* OR initial amoxicillin OR initial antibacterial OR initial antibiotic OR initial antibiotics OR initial antimicrobial OR initial course OR initial drug OR initial penicillin OR initial therap* OR initial treatment OR initial treatments OR judicious OR "length of therap*" OR "length of treatment" OR long course OR long courses OR long drug OR "long drugs" OR long regimens OR long term amoxicillin OR long term antibiotic OR long term antibiotics OR long term penicillin OR long term therap* OR long term treatment OR long term treatments OR long treatment OR longer course OR longer courses OR longer regimens OR longer treatment OR longer treatments OR mis-diagnos* OR misdiagnos* OR missed diagnos* OR month course OR month treatment OR monthly courses OR "months of oral*" OR "months of therap*" OR "months of treatment" OR months treatment OR National Institute for Health and Care Excellence OR national recommendation OR national recommendations OR NICE recommend* OR nonguideline OR "number of courses" OR "number of prescribed" OR "number of prescriptions" OR observation OR one course OR one prescription OR optimal length* OR oral course OR oral regimen OR oral regimens OR over-diagnos* OR over-prescrib* OR over-prescription OR over-prescriptions OR overdiagnos* OR overprescrib* OR overprescription OR parent decision* OR parents decision* OR patient decision* OR patients decision* OR penicillin course OR penicillin courses OR prescribed daily OR prescribing habits OR prescribing practice OR prescribing practices OR prescribing rate OR prescribing rates OR prescription habits OR prescription length* OR prescription practice OR prescription practices OR prescription rate OR prescription rates OR prescriptive practice OR prescriptive practices OR prolonged antibiotic OR prolonged course OR prolonged courses OR prolonged medication OR prolonged penicillin OR prolonged therap* OR prolonged treatment OR proper diagnos* OR properly diagnos* OR prudent OR "rate of antibiotic" OR "rate of antibiotics" OR "rate of prescribing" OR "rate of prescription" OR "rates of antibiotic" OR "rates of antibiotics" OR "rates of prescribing" OR

"regimens of antibiotic" OR "regimens of antibiotics" OR safety net antibiotic OR second course OR second prescription OR second treatment OR shared decision* OR short antibiotic OR "short antibiotics" OR short course OR short courses OR short regimen OR short regimens OR short term antibiotic OR short term antibiotics OR short term oral* OR short term penicillin OR short term treatment OR short term treatments OR shortened antimicrobial OR shortened course OR shortened courses OR shortened therap* OR shortened treatment OR shorter antibiotic OR shorter course OR shorter courses OR shorter prescription OR shorter regimen OR shorter regimens OR shorter treatment OR standard courses OR standard regimen OR standard regimens OR steward* OR therapeutic recommendation OR therapeutic recommendations OR therapy courses OR therapy recommendation OR therapy regimens OR therapy recommendations OR three courses OR treatment course OR treatment courses OR treatment day OR treatment days OR treatment length* OR treatment recommendation OR treatment recommendations OR treatment regimen OR treatment regimens OR treatment time OR two courses OR two regimens OR two treatments OR under-diagnos* OR under-prescrib* OR under-prescription OR under-prescriptions OR underdiagnos* OR underprescrib* OR underprescription OR unnecessary OR wait* OR watchful OR watching OR week antibiotic OR week course OR weeks course OR "weeks of antibiotic" OR withheld OR withhold*) OR AB ("1 course" OR "2 courses" OR "3 courses" OR "4 courses" OR "5 courses" OR AAP recommend* OR active surveillance OR administration schedule OR administration schedules OR American Academy of Pediatrics OR amoxicillin course OR antibiotic course OR antibiotic courses OR antibiotic days OR "antibiotics longer" OR appropriate* OR complete course OR complete courses OR complete drug OR "complete drugs" OR completed course OR "completed drug" OR "completed oral*" OR completed therap* OR completed treatment OR "completing antibiotic" OR completing therap* OR completing treatment OR course amoxycillin OR course antibacterial OR course antibiotic OR course antibiotics OR course antimicrobial OR course antimicrobials OR course daily OR course length* OR "course of amoxicillin" OR "course of amoxycillin" OR "course of antibacterial" OR "course of antibiotic"

OR "course of antibiotics" OR "course of antimicrobial" OR "course of antimicrobials" OR "course of oral*" OR "course of penicillin" OR "course of therap*" OR "course of treatment" OR course oral* OR course regimen OR course regimens OR "courses longer" OR "courses of antibiotic" OR "courses of antibiotics" OR "courses of antimicrobial" OR "courses of antimicrobials" OR "courses of oral*" OR "courses of therap*" OR "courses of treatment" OR courses prescribed OR daily course OR daily oral* OR daily treatment OR day amoxicillin OR day amoxycillin OR day antibiotic OR day course OR day oral* OR day penicillin OR day regimen OR day regimens OR day therap* OR day treatment OR days course OR "days of amoxicillin" OR "days of amoxycillin" OR "days of antibiotic" OR "days of antibiotics" OR "days of oral*" OR "days of therap*" OR "days of treatment" OR days therap* OR days treatment OR delay* OR diagnoses criteria OR diagnosing ability OR diagnosing criteria OR diagnosis abilities OR diagnosis ability OR diagnosis behavior* OR diagnosis behaviour* OR diagnosis criteria OR diagnosis skill OR diagnosis skills OR diagnosis validity OR diagnostic abilities OR diagnostic ability OR diagnostic behavior* OR diagnostic behaviour* OR diagnostic criteria OR diagnostic criterion OR diagnostic skill OR diagnostic skills OR diagnostic validity OR diagnostical criteria OR diagnostical criterion OR diagnostical validity OR "difficult to diagnose" OR duration OR durations OR expectant OR extended treatment OR extending treatment OR failed diagnos* OR "failure to diagnos*" OR first course OR first prescription OR five courses OR four courses OR fourth course OR full course OR full courses OR full treatment OR full treatments OR guidance OR guideline OR guidelines OR immediacy OR immediate OR immediately OR improper diagnos* OR inappropriate* OR initial amoxicillin OR initial antibacterial OR initial antibiotic OR initial antibiotics OR initial antimicrobial OR initial course OR initial drug OR initial penicillin OR initial therap* OR initial treatment OR initial treatments OR judicious OR "length of therap*" OR "length of treatment" OR long course OR long courses OR long drug OR "long drugs" OR long regimens OR long term amoxicillin OR long term antibiotic OR long term antibiotics OR long term penicillin OR long term therap* OR long term treatment OR long term treatments OR long treatment OR longer course OR longer

courses OR longer regimens OR longer treatment OR longer treatments OR mis-diagnos* OR misdiagnos* OR missed diagnos* OR month course OR month treatment OR monthly courses OR "months of oral*" OR "months of therap*" OR "months of treatment" OR months treatment OR National Institute for Health and Care Excellence OR national recommendation OR national recommendations OR NICE recommend* OR nonguideline OR "number of courses" OR "number of prescribed" OR "number of prescriptions" OR observation OR one course OR one prescription OR optimal length* OR oral course OR oral regimen OR oral regimens OR over-diagnos* OR over-prescrib* OR over-prescription OR over-prescriptions OR overdiagnos* OR overprescrib* OR overprescription OR parent decision* OR parents decision* OR patient decision* OR patients decision* OR penicillin course OR penicillin courses OR prescribed daily OR prescribing habits OR prescribing practice OR prescribing practices OR prescribing rate OR prescribing rates OR prescription habits OR prescription length* OR prescription practice OR prescription practices OR prescription rate OR prescription rates OR prescriptive practice OR prescriptive practices OR prolonged antibiotic OR prolonged course OR prolonged courses OR prolonged medication OR prolonged penicillin OR prolonged therap* OR prolonged treatment OR proper diagnos* OR properly diagnos* OR prudent OR "rate of antibiotic" OR "rate of antibiotics" OR "rate of prescribing" OR "rate of prescription" OR "rates of antibiotic" OR "rates of antibiotics" OR "rates of prescribing" OR "regimens of antibiotic" OR "regimens of antibiotics" OR safety net antibiotic OR second course OR second prescription OR second treatment OR shared decision* OR short antibiotic OR "short antibiotics" OR short course OR short courses OR short regimen OR short regimens OR short term antibiotic OR short term antibiotics OR short term oral* OR short term penicillin OR short term treatment OR short term treatments OR shortened antimicrobial OR shortened course OR shortened courses OR shortened therap* OR shortened treatment OR shorter antibiotic OR shorter course OR shorter courses OR shorter prescription OR shorter regimen OR shorter regimens OR shorter treatment OR standard courses OR standard regimen OR standard regimens OR steward* OR therapeutic recommendation OR therapeutic

recommendations OR therapy courses OR therapy recommendation OR therapy regimens OR therapy recommendations OR three courses OR treatment course OR treatment courses OR treatment day OR treatment days OR treatment length* OR treatment recommendation OR treatment recommendations OR treatment regimen OR treatment regimens OR treatment time OR two courses OR two regimens OR two treatments OR under-diagnos* OR under-prescrib* OR under-prescription OR under-prescriptions OR underdiagnos* OR underprescrib* OR underprescription OR unnecessary OR wait* OR watchful OR watching OR week antibiotic OR week course OR weeks course OR "weeks of antibiotic" OR withheld OR withhold*) OR ((TI diagnos* OR AB diagnos*) AND (TI (accurac* OR accurate* OR blind spot OR blind spots OR capabilities OR capability OR certainties OR certainty OR correct OR correctly OR difficulties OR difficulty OR erroneous OR error OR errors OR false OR faulty OR flawed OR imprecise* OR imprecision OR inaccurac* OR inaccurate* OR incorrect OR incorrectly OR judgment OR judgments OR mistake* OR precise* OR precision OR strict criteria OR stringent criteria OR uncertainties OR uncertainty OR wrong) OR AB (accurac* OR accurate* OR blind spot OR blind spots OR capabilities OR capability OR certainties OR certainty OR correct OR correctly OR difficulties OR difficulty OR erroneous OR error OR errors OR false OR faulty OR flawed OR imprecise* OR imprecision OR inaccurac* OR inaccurate* OR incorrect OR incorrectly OR judgment OR judgments OR mistake* OR precise* OR precision OR strict criteria OR stringent criteria OR uncertainties OR uncertainty OR wrong))) OR ((MH "Prescriptions, Drug+" OR TI (dispensing OR prescribe* OR prescribing OR prescription OR prescriptions OR prescriptive) OR AB (dispensing OR prescribe* OR prescribing OR prescription OR prescriptions OR prescriptive)) AND (MH "Practice Patterns" OR TI (annual OR annually OR behavior* OR behaviour* OR improper OR improperly OR over-consum* OR over-use* OR overconsum* OR overuse* OR pattern OR patterns OR proper OR properly OR trend OR trends OR under-consum* OR under-use* OR underconsum* OR underuse*) OR AB (annual OR annually OR behavior* OR behaviour* OR improper OR improperly OR over-consum* OR over-use* OR overconsum* OR overuse* OR pattern OR patterns

OR proper OR properly OR trend OR trends OR under-consum* OR under-use* OR underconsum* OR underuse*)))) AND (MH "Amoxicillin" OR MH "Antibiotics+" OR MH "Antiinfective Agents" OR MH "Azithromycin" OR MH "Ceftriaxone" OR MH "Cefotaxime+" OR MH "Ciprofloxacin" OR MH "Penicillins+" OR TI (amoxicillin* OR amoxicilline* OR amoxy* OR amoxycillin* OR amoxycilline* OR anti-bacteria* OR anti-biotic* OR anti-infect* OR anti-microbial* OR anti-mycobacteria* OR antibacteria* OR antibiotic* OR antiinfect* OR antimicrobial* OR antimycobacteria* OR azithromycin OR azitromicina OR azythromycin OR bacteriocid* OR cef triaxone OR cefatriaxone OR cefdinir OR cefotaxim OR cefotaxime OR cefotriaxone OR ceftriaxon OR ceftriaxone OR cephotaxim OR ciprofloxacin OR microbicide OR microbicides OR penicillin OR penicillins) OR AB (amoxicillin* OR amoxicilline* OR amoxy* OR amoxycillin* OR amoxycilline* OR anti-bacteria* OR anti-biotic* OR anti-infect* OR anti-microbial* OR anti-mycobacteria* OR antibacteria* OR antibiotic* OR antiinfect* OR antimicrobial* OR antimycobacteria* OR azithromycin OR azitromicina OR azythromycin OR bacteriocid* OR cef triaxone OR cefatriaxone OR cefdinir OR cefotaxim OR cefotaxime OR cefotriaxone OR ceftriaxon OR ceftriaxone OR cephotaxim OR ciprofloxacin OR microbicide OR microbicides OR penicillin OR penicillins)) AND DT 20000101-20230630 NOT (MH "Adult+" NOT (MH "Adolescence+" OR MH "Child+" OR MH "Pediatrics+"))

Embase (Elsevier): http://www.library.vanderbilt.edu/eres?id=1770

Date Last Searched: 7/19/2023

Results: 2,557

Limits: Limited to children, Publication dates 2000-2023, Excluded conference/meeting abstracts

('acute otitis media'/exp OR 'infected middle ear':ab,kw,ti OR 'infected middle ears':ab,kw,ti OR 'infectious middle ear':ab,kw,ti OR 'inflamed middle ear':ab,kw,ti OR 'inflammation of middle

ear':ab,kw,ti OR 'inflammation of middle ears':ab,kw,ti OR 'inflammation of the middle ear':ab,kw,ti OR 'inflammation of the middle ears':ab,kw,ti OR 'inflammatory middle ear':ab,kw,ti OR 'inflammatory middle ears':ab,kw,ti OR 'media otitis':ab,kw,ti OR 'middle ear infect*':ab,kw,ti OR 'middle ear inflam*':ab,kw,ti OR 'middle otitis':ab,kw,ti OR 'otitis media*':ab,kw,ti OR 'otitis middle':ab,kw,ti) AND ('antimicrobial stewardship'/exp OR 'diagnostic accuracy'/exp OR 'diagnostic error'/exp OR 'observation'/exp OR 'patient decision making'/exp OR 'practice guideline'/exp OR 'prescribing error'/exp OR 'shared decision making'/exp OR 'treatment duration'/exp OR 'unnecessary procedure'/exp OR 'watchful waiting'/exp OR '1 course':ab,kw,ti OR '2 courses':ab,kw,ti OR '3 courses':ab,kw,ti OR '4 courses':ab,kw,ti OR '5 courses':ab,kw,ti OR 'aap recommend*':ab,kw,ti OR 'active surveillance':ab,kw,ti OR 'administration schedule':ab,kw,ti OR 'administration schedules':ab,kw,ti OR 'american academy of pediatrics':ab,kw,ti OR 'amoxicillin course':ab,kw,ti OR 'antibiotic course':ab,kw,ti OR 'antibiotic courses':ab,kw,ti OR 'antibiotic days':ab,kw,ti OR 'antibiotics longer':ab,kw,ti OR appropriate*:ab,kw,ti OR 'complete course':ab,kw,ti OR 'complete courses':ab,kw,ti OR 'complete drug':ab,kw,ti OR 'complete drugs':ab,kw,ti OR 'completed course':ab,kw,ti OR 'completed drug':ab,kw,ti OR 'completed oral*':ab,kw,ti OR 'completed therap*':ab,kw,ti OR 'completed treatment':ab,kw,ti OR 'completing antibiotic':ab,kw,ti OR 'completing therap*':ab,kw,ti OR 'completing treatment':ab,kw,ti OR 'course amoxycillin':ab,kw,ti OR 'course antibacterial':ab,kw,ti OR 'course antibiotic':ab,kw,ti OR 'course antibiotics':ab,kw,ti OR 'course antimicrobial':ab,kw,ti OR 'course antimicrobials':ab,kw,ti OR 'course daily':ab,kw,ti OR 'course length*':ab,kw,ti OR 'course of amoxicillin':ab,kw,ti OR 'course of amoxycillin':ab,kw,ti OR 'course of antibacterial':ab,kw,ti OR 'course of antibiotic':ab,kw,ti OR 'course of antibiotics':ab,kw,ti OR 'course of antimicrobial':ab,kw,ti OR 'course of antimicrobials':ab,kw,ti OR 'course of oral*':ab,kw,ti OR 'course of penicillin':ab,kw,ti OR 'course of therap*':ab,kw,ti OR 'course of treatment':ab,kw,ti OR 'course oral*':ab,kw,ti OR 'course regimen':ab,kw,ti OR 'course regimens':ab,kw,ti OR 'courses longer':ab,kw,ti OR 'courses of antibiotic':ab,kw,ti OR 'courses of antibiotics':ab,kw,ti OR

'courses of antimicrobial':ab,kw,ti OR 'courses of antimicrobials':ab,kw,ti OR 'courses of oral*':ab,kw,ti OR 'courses of therap*':ab,kw,ti OR 'courses of treatment':ab,kw,ti OR 'courses prescribed':ab,kw,ti OR 'daily course':ab,kw,ti OR 'daily oral*':ab,kw,ti OR 'daily treatment':ab,kw,ti OR 'day amoxicillin':ab,kw,ti OR 'day amoxycillin':ab,kw,ti OR 'day antibiotic':ab,kw,ti OR 'day course':ab,kw,ti OR 'day oral*':ab,kw,ti OR 'day penicillin':ab,kw,ti OR 'day regimen':ab,kw,ti OR 'day regimens':ab,kw,ti OR 'day therap*':ab,kw,ti OR 'day treatment':ab,kw,ti OR 'days course':ab,kw,ti OR 'days of amoxicillin':ab,kw,ti OR 'days of amoxycillin':ab,kw,ti OR 'days of antibiotic':ab,kw,ti OR 'days of antibiotics':ab,kw,ti OR 'days of oral*':ab,kw,ti OR 'days of therap*':ab,kw,ti OR 'days of treatment':ab,kw,ti OR 'days therap*':ab,kw,ti OR 'days treatment':ab,kw,ti OR delay*:ab,kw,ti OR 'diagnoses criteria':ab,kw,ti OR 'diagnosing ability':ab,kw,ti OR 'diagnosing criteria':ab,kw,ti OR 'diagnosis abilities':ab,kw,ti OR 'diagnosis ability':ab,kw,ti OR 'diagnosis behavior*':ab,kw,ti OR 'diagnosis behaviour*':ab,kw,ti OR 'diagnosis criteria':ab,kw,ti OR 'diagnosis skill':ab,kw,ti OR 'diagnosis skills':ab,kw,ti OR 'diagnosis validity':ab,kw,ti OR 'diagnostic abilities':ab,kw,ti OR 'diagnostic ability':ab,kw,ti OR 'diagnostic behavior*':ab,kw,ti OR 'diagnostic behaviour*':ab,kw,ti OR 'diagnostic criteria':ab,kw,ti OR 'diagnostic criterion':ab,kw,ti OR 'diagnostic skill':ab,kw,ti OR 'diagnostic skills':ab,kw,ti OR 'diagnostic validity':ab,kw,ti OR 'diagnostical criteria':ab,kw,ti OR 'diagnostical criterion':ab,kw,ti OR 'diagnostical validity':ab,kw,ti OR 'difficult to diagnose':ab,kw,ti OR duration:ab,kw,ti OR durations:ab,kw,ti OR expectant:ab,kw,ti OR 'extended treatment':ab,kw,ti OR 'extending treatment':ab,kw,ti OR 'failed diagnos*':ab,kw,ti OR 'failure to diagnos*':ab,kw,ti OR 'first course':ab,kw,ti OR 'first prescription':ab,kw,ti OR 'five courses':ab,kw,ti OR 'four courses':ab,kw,ti OR 'fourth course':ab,kw,ti OR 'full course':ab,kw,ti OR 'full courses':ab,kw,ti OR 'full treatment':ab,kw,ti OR 'full treatments':ab,kw,ti OR guidance:ab,kw,ti OR guideline:ab,kw,ti OR guidelines:ab,kw,ti OR immediacy:ab,kw,ti OR immediate:ab,kw,ti OR immediately:ab,kw,ti OR 'improper diagnos*':ab,kw,ti OR inappropriate*:ab,kw,ti OR 'initial amoxicillin':ab,kw,ti OR 'initial antibacterial':ab,kw,ti OR 'initial antibiotic':ab,kw,ti OR 'initial antibiotics':ab,kw,ti OR 'initial

antimicrobial':ab,kw,ti OR 'initial course':ab,kw,ti OR 'initial drug':ab,kw,ti OR 'initial penicillin':ab,kw,ti OR 'initial therap*':ab,kw,ti OR 'initial treatment':ab,kw,ti OR 'initial treatments':ab,kw,ti OR judicious:ab,kw,ti OR 'length of therap*':ab,kw,ti OR 'length of treatment':ab,kw,ti OR 'long course':ab,kw,ti OR 'long courses':ab,kw,ti OR 'long drug':ab,kw,ti OR 'long drugs':ab,kw,ti OR 'long regimens':ab,kw,ti OR 'long term amoxicillin':ab,kw,ti OR 'long term antibiotic':ab,kw,ti OR 'long term antibiotics':ab,kw,ti OR 'long term penicillin':ab,kw,ti OR 'long term therap*':ab,kw,ti OR 'long term treatment':ab,kw,ti OR 'long term treatments':ab,kw,ti OR 'long treatment':ab,kw,ti OR 'longer course':ab,kw,ti OR 'longer courses':ab,kw,ti OR 'longer regimens':ab,kw,ti OR 'longer treatment':ab,kw,ti OR 'longer treatments':ab,kw,ti OR 'mis diagnos*':ab,kw,ti OR misdiagnos*:ab,kw,ti OR 'missed diagnos*':ab,kw,ti OR 'month course':ab,kw,ti OR 'month treatment':ab,kw,ti OR 'monthly courses':ab,kw,ti OR 'months of oral*':ab,kw,ti OR 'months of therap*':ab,kw,ti OR 'months of treatment':ab,kw,ti OR 'months treatment':ab,kw,ti OR 'national institute for health and care excellence':ab,kw,ti OR 'national recommendation':ab,kw,ti OR 'national recommendations':ab,kw,ti OR 'nice recommend*':ab,kw,ti OR nonguideline:ab,kw,ti OR 'number of courses':ab,kw,ti OR 'number of prescribed':ab,kw,ti OR 'number of prescriptions':ab,kw,ti OR observation:ab,kw,ti OR 'one course':ab,kw,ti OR 'one prescription':ab,kw,ti OR 'optimal length*':ab,kw,ti OR 'oral course':ab,kw,ti OR 'oral regimen':ab,kw,ti OR 'oral regimens':ab,kw,ti OR 'over diagnos*':ab,kw,ti OR 'over prescrib*':ab,kw,ti OR 'over prescription':ab,kw,ti OR 'over prescriptions':ab,kw,ti OR overdiagnos*:ab,kw,ti OR overprescrib*:ab,kw,ti OR overprescription:ab,kw,ti OR 'parent decision*':ab,kw,ti OR 'parents decision*':ab,kw,ti OR 'patient decision*':ab,kw,ti OR 'patients decision*':ab,kw,ti OR 'penicillin course':ab,kw,ti OR 'penicillin courses':ab,kw,ti OR 'prescribed daily':ab,kw,ti OR 'prescribing habits':ab,kw,ti OR 'prescribing practice':ab,kw,ti OR 'prescribing practices':ab,kw,ti OR 'prescribing rate':ab,kw,ti OR 'prescribing rates':ab,kw,ti OR 'prescription habits':ab,kw,ti OR 'prescription length*':ab,kw,ti OR 'prescription practice':ab,kw,ti OR 'prescription

practices':ab,kw,ti OR 'prescription rate':ab,kw,ti OR 'prescription rates':ab,kw,ti OR 'prescriptive practice':ab,kw,ti OR 'prescriptive practices':ab,kw,ti OR 'prolonged antibiotic':ab,kw,ti OR 'prolonged course':ab,kw,ti OR 'prolonged courses':ab,kw,ti OR 'prolonged medication':ab,kw,ti OR 'prolonged penicillin':ab,kw,ti OR 'prolonged therap*':ab,kw,ti OR 'prolonged treatment':ab,kw,ti OR 'proper diagnos*':ab,kw,ti OR 'properly diagnos*':ab,kw,ti OR prudent:ab,kw,ti OR 'rate of antibiotic':ab,kw,ti OR 'rate of antibiotics':ab,kw,ti OR 'rate of prescribing':ab,kw,ti OR 'rate of prescription':ab,kw,ti OR 'rates of antibiotic':ab,kw,ti OR 'rates of antibiotics':ab,kw,ti OR 'rates of prescribing':ab,kw,ti OR 'regimens of antibiotic':ab,kw,ti OR 'regimens of antibiotics':ab,kw,ti OR 'safety net antibiotic':ab,kw,ti OR 'second course':ab,kw,ti OR 'second prescription':ab,kw,ti OR 'second treatment':ab,kw,ti OR 'shared decision*':ab,kw,ti OR 'short antibiotic':ab,kw,ti OR 'short antibiotics':ab,kw,ti OR 'short course':ab,kw,ti OR 'short courses':ab,kw,ti OR 'short regimen':ab,kw,ti OR 'short regimens':ab,kw,ti OR 'short term antibiotic':ab,kw,ti OR 'short term antibiotics':ab,kw,ti OR 'short term oral*':ab,kw,ti OR 'short term penicillin':ab,kw,ti OR 'short term treatment':ab,kw,ti OR 'short term treatments':ab,kw,ti OR 'shortened antimicrobial':ab,kw,ti OR 'shortened course':ab,kw,ti OR 'shortened courses':ab,kw,ti OR 'shortened therap*':ab,kw,ti OR 'shortened treatment':ab,kw,ti OR 'shorter antibiotic':ab,kw,ti OR 'shorter course':ab,kw,ti OR 'shorter courses':ab,kw,ti OR 'shorter prescription':ab,kw,ti OR 'shorter regimen':ab,kw,ti OR 'shorter regimens':ab,kw,ti OR 'shorter treatment':ab,kw,ti OR 'standard courses':ab,kw,ti OR 'standard regimen':ab,kw,ti OR 'standard regimens':ab,kw,ti OR steward*:ab,kw,ti OR 'therapeutic recommendation':ab,kw,ti OR 'therapeutic recommendations':ab,kw,ti OR 'therapy courses':ab,kw,ti OR 'therapy recommendation':ab,kw,ti OR 'therapy regimens':ab,kw,ti OR 'therapy recommendations':ab,kw,ti OR 'three courses':ab,kw,ti OR 'treatment course':ab,kw,ti OR 'treatment courses':ab,kw,ti OR 'treatment day':ab,kw,ti OR 'treatment days':ab,kw,ti OR 'treatment length*':ab,kw,ti OR 'treatment recommendation':ab,kw,ti OR 'treatment recommendations':ab,kw,ti OR 'treatment regimen':ab,kw,ti OR 'treatment regimens':ab,kw,ti OR 'treatment time':ab,kw,ti OR 'two courses':ab,kw,ti

OR 'two regimens':ab,kw,ti OR 'two treatments':ab,kw,ti OR 'under diagnos*':ab,kw,ti OR 'under prescrib*':ab,kw,ti OR 'under prescription':ab,kw,ti OR 'under prescriptions':ab,kw,ti OR underdiagnos*:ab,kw,ti OR underprescrib*:ab,kw,ti OR underprescription:ab,kw,ti OR unnecessary:ab,kw,ti OR wait*:ab,kw,ti OR watchful:ab,kw,ti OR watching:ab,kw,ti OR 'week antibiotic':ab,kw,ti OR 'week course':ab,kw,ti OR 'weeks course':ab,kw,ti OR 'weeks of antibiotic':ab,kw,ti OR withheld:ab,kw,ti OR withhold*:ab,kw,ti OR (diagnos*:ab,kw,ti AND (accurac*:ab,kw,ti OR accurate*:ab,kw,ti OR 'blind spot':ab,kw,ti OR 'blind spots':ab,kw,ti OR capabilities:ab,kw,ti OR capability:ab,kw,ti OR certainties:ab,kw,ti OR certainty:ab,kw,ti OR correct:ab,kw,ti OR correctly:ab,kw,ti OR difficulties:ab,kw,ti OR difficulty:ab,kw,ti OR erroneous:ab,kw,ti OR error:ab,kw,ti OR errors:ab,kw,ti OR false:ab,kw,ti OR faulty:ab,kw,ti OR flawed:ab,kw,ti OR imprecise*:ab,kw,ti OR imprecision:ab,kw,ti OR inaccurac*:ab,kw,ti OR inaccurate*:ab,kw,ti OR incorrect:ab,kw,ti OR incorrectly:ab,kw,ti OR judgment:ab,kw,ti OR judgments:ab,kw,ti OR mistake*:ab,kw,ti OR precise*:ab,kw,ti OR precision:ab,kw,ti OR 'strict criteria':ab,kw,ti OR 'stringent criteria':ab,kw,ti OR uncertainties:ab,kw,ti OR uncertainty:ab,kw,ti OR wrong:ab,kw,ti)) OR (('prescription'/exp OR dispensing:ab,kw,ti OR prescribe*:ab,kw,ti OR prescribing:ab,kw,ti OR prescription:ab,kw,ti OR prescriptions:ab,kw,ti OR prescriptive:ab,kw,ti) AND ('clinical practice'/exp OR annual:ab,kw,ti OR annually:ab,kw,ti OR behavior*:ab,kw,ti OR behaviour*:ab,kw,ti OR improper:ab,kw,ti OR improperly:ab,kw,ti OR 'over consum*':ab,kw,ti OR 'over use*':ab,kw,ti OR overconsum*:ab,kw,ti OR overuse*:ab,kw,ti OR pattern:ab,kw,ti OR patterns:ab,kw,ti OR proper:ab,kw,ti OR properly:ab,kw,ti OR trend:ab,kw,ti OR trends:ab,kw,ti OR 'under consum*':ab,kw,ti OR 'under use*':ab,kw,ti OR underconsum*:ab,kw,ti OR underuse*:ab,kw,ti))) AND ('amoxicillin'/exp OR 'antibiotic agent'/exp OR 'antiinfective agent'/de OR 'azithromycin'/exp OR 'cefdinir'/exp OR 'ceftriaxone'/exp OR 'cefotaxime'/exp OR 'ciprofloxacin'/exp OR 'penicillin derivative'/exp OR amoxicillin*:ab,kw,ti OR amoxicilline*:ab,kw,ti OR amoxy*:ab,kw,ti OR amoxycillin*:ab,kw,ti OR amoxycilline*:ab,kw,ti OR 'anti bacteria*':ab,kw,ti OR 'anti biotic*':ab,kw,ti OR

'anti infect*':ab,kw,ti OR 'anti microbial*':ab,kw,ti OR 'anti mycobacteria*':ab,kw,ti OR antibacteria*:ab,kw,ti OR antibiotic*:ab,kw,ti OR antiinfect*:ab,kw,ti OR antimicrobial*:ab,kw,ti OR antimycobacteria*:ab,kw,ti OR azithromycin:ab,kw,ti OR azitromicina:ab,kw,ti OR azythromycin:ab,kw,ti OR bacteriocid*:ab,kw,ti OR 'cef triaxone':ab,kw,ti OR cefatriaxone:ab,kw,ti OR cefdinir:ab,kw,ti OR cefotaxim:ab,kw,ti OR cefotaxime:ab,kw,ti OR cefotriaxone:ab,kw,ti OR ceftriaxon:ab,kw,ti OR ceftriaxone:ab,kw,ti OR cephotaxim:ab,kw,ti OR ciprofloxacin:ab,kw,ti OR microbicide:ab,kw,ti OR microbicides:ab,kw,ti OR penicillin:ab,kw,ti OR penicillins:ab,kw,ti) AND (2000:py OR 2001:py OR 2002:py OR 2003:py OR 2004:py OR 2005:py OR 2006:py OR 2007:py OR 2008:py OR 2009:py OR 2010:py OR 2011:py OR 2012:py OR 2013:py OR 2014:py OR 2015:py OR 2016:py OR 2017:py OR 2018:py OR 2019:py OR 2020:py OR 2021:py OR 2022:py OR 2023:py) NOT ('adult'/exp NOT ('juvenile'/exp OR 'pediatrics'/exp)) NOT 'conference abstract'/it

Web of Science, Core Collection (Clarivate): http://www.library.vanderbilt.edu/eres?id=1241

Date Last Searched: 7/19/2023

Results: 1,857

Limits: Publication dates 2000-2023, Excluded conference/meeting abstracts

TS=("infected middle ear" OR "infected middle ears" OR "infectious middle ear" OR "inflamed middle ear" OR "inflammation of middle ear" OR "inflammation of middle ears" OR "inflammation of the middle ear" OR "inflammation of the middle ears" OR "inflammatory middle ear" OR "inflammatory middle ears" OR "media otitis" OR "middle ear infect*" OR "middle ear inflam*" OR "middle otitis" OR "otitis media*" OR "otitis middle") AND TS=("1 course" OR "2 courses" OR "3 courses" OR "4 courses" OR "5 courses" OR "AAP recommend*" OR "active surveillance" OR "administration schedule" OR "administration schedules" OR "American Academy of Pediatrics" OR "amoxicillin course" OR "antibiotic course" OR

"antibiotic courses" OR "antibiotic days" OR "antibiotics longer" OR appropriate* OR "complete course" OR "complete courses" OR "complete drug" OR "complete drugs" OR "completed course" OR "completed drug" OR "completed oral*" OR "completed therap*" OR "completed treatment" OR "completing antibiotic" OR "completing therap*" OR "completing treatment" OR "course amoxycillin" OR "course antibacterial" OR "course antibiotic" OR "course antibiotics" OR "course antimicrobial" OR "course antimicrobials" OR "course daily" OR "course length*" OR "course of amoxicillin" OR "course of amoxycillin" OR "course of antibacterial" OR "course of antibiotic" OR "course of antibiotics" OR "course of antimicrobial" OR "course of antimicrobials" OR "course of oral*" OR "course of penicillin" OR "course of therap*" OR "course of treatment" OR "course oral*" OR "course regimen" OR "course regimens" OR "courses longer" OR "courses of antibiotic" OR "courses of antibiotics" OR "courses of antimicrobial" OR "courses of antimicrobials" OR "courses of oral*" OR "courses of therap*" OR "courses of treatment" OR "courses prescribed" OR "daily course" OR "daily oral*" OR "daily treatment" OR "day amoxicillin" OR "day amoxycillin" OR "day antibiotic" OR "day course" OR "day oral*" OR "day penicillin" OR "day regimen" OR "day regimens" OR "day therap*" OR "day treatment" OR "days course" OR "days of amoxicillin" OR "days of amoxycillin" OR "days of antibiotic" OR "days of antibiotics" OR "days of oral*" OR "days of therap*" OR "days of treatment" OR "days therap*" OR "days treatment" OR delay* OR "diagnoses criteria" OR "diagnosing ability" OR "diagnosing criteria" OR "diagnosis abilities" OR "diagnosis ability" OR "diagnosis behavior*" OR "diagnosis behaviour*" OR "diagnosis criteria" OR "diagnosis skill" OR "diagnosis skills" OR "diagnosis validity" OR "diagnostic abilities" OR "diagnostic ability" OR "diagnostic behavior*" OR "diagnostic behaviour*" OR "diagnostic criteria" OR "diagnostic criterion" OR "diagnostic skill" OR "diagnostic skills" OR "diagnostic validity" OR "diagnostical criteria" OR "diagnostical criterion" OR "diagnostical validity" OR "difficult to diagnose" OR duration OR durations OR expectant OR "extended treatment" OR "extending treatment" OR "failed diagnos*" OR "failure to diagnos*" OR "first course" OR "first prescription" OR "five courses" OR "four courses" OR

"fourth course" OR "full course" OR "full courses" OR "full treatment" OR "full treatments" OR guidance OR guideline OR guidelines OR immediacy OR immediate OR immediately OR "improper diagnos*" OR inappropriate* OR "initial amoxicillin" OR "initial antibacterial" OR "initial antibiotic" OR "initial antibiotics" OR "initial antimicrobial" OR "initial course" OR "initial drug" OR "initial penicillin" OR "initial therap*" OR "initial treatment" OR "initial treatments" OR judicious OR "length of therap*" OR "length of treatment" OR "long course" OR "long courses" OR "long drug" OR "long drugs" OR "long regimens" OR "long term amoxicillin" OR "long term antibiotic" OR "long term antibiotics" OR "long term penicillin" OR "long term therap*" OR "long term treatment" OR "long term treatments" OR "long treatment" OR "longer course" OR "longer courses" OR "longer regimens" OR "longer treatment" OR "longer treatments" OR mis-diagnos* OR misdiagnos* OR "missed diagnos*" OR "month course" OR "month treatment" OR "monthly courses" OR "months of oral*" OR "months of therap*" OR "months of treatment" OR "months treatment" OR "National Institute for Health and Care Excellence" OR "national recommendation" OR "national recommendations" OR "NICE recommend*" OR nonguideline OR "number of courses" OR "number of prescribed" OR "number of prescriptions" OR observation OR "one course" OR "one prescription" OR "optimal length*" OR "oral course" OR "oral regimen" OR "oral regimens" OR over-diagnos* OR over-prescrib* OR over-prescription OR over-prescriptions OR overdiagnos* OR overprescrib* OR overprescription OR "parent decision*" OR "parents decision*" OR "patient decision*" OR "patients decision*" OR "penicillin course" OR "penicillin courses" OR "prescribed daily" OR "prescribing habits" OR "prescribing practice" OR "prescribing practices" OR "prescribing rate" OR "prescribing rates" OR "prescription habits" OR "prescription length*" OR "prescription practice" OR "prescription practices" OR "prescription rate" OR "prescription rates" OR "prescriptive practice" OR "prescriptive practices" OR "prolonged antibiotic" OR "prolonged course" OR "prolonged courses" OR "prolonged medication" OR "prolonged penicillin" OR "prolonged therap*" OR "prolonged treatment" OR "proper diagnos*" OR "properly diagnos*" OR prudent OR "rate of antibiotic" OR "rate of antibiotics" OR

"rate of prescribing" OR "rate of prescription" OR "rates of antibiotic" OR "rates of antibiotics" OR "rates of prescribing" OR "regimens of antibiotic" OR "regimens of antibiotics" OR "safety net antibiotic" OR "second course" OR "second prescription" OR "second treatment" OR "shared decision*" OR "short antibiotic" OR "short antibiotics" OR "short course" OR "short courses" OR "short regimen" OR "short regimens" OR "short term antibiotic" OR "short term antibiotics" OR "short term oral*" OR "short term penicillin" OR "short term treatment" OR "short term treatments" OR "shortened antimicrobial" OR "shortened course" OR "shortened courses" OR "shortened therap*" OR "shortened treatment" OR "shorter antibiotic" OR "shorter course" OR "shorter courses" OR "shorter prescription" OR "shorter regimen" OR "shorter regimens" OR "shorter treatment" OR "standard courses" OR "standard regimen" OR "standard regimens" OR steward* OR "therapeutic recommendation" OR "therapeutic recommendations" OR "therapy courses" OR "therapy recommendation" OR "therapy regimens" OR "therapy recommendations" OR "three courses" OR "treatment course" OR "treatment courses" OR "treatment day" OR "treatment days" OR "treatment length*" OR "treatment recommendation" OR "treatment recommendations" OR "treatment regimen" OR "treatment regimens" OR "treatment time" OR "two courses" OR "two regimens" OR "two treatments" OR under-diagnos* OR under-prescrib* OR under-prescription OR under-prescriptions OR underdiagnos* OR underprescrib* OR underprescription OR unnecessary OR wait* OR watchful OR watching OR "week antibiotic" OR "week course" OR "weeks course" OR "weeks of antibiotic" OR withheld OR withhold* OR (diagnos* AND (accurac* OR accurate* OR "blind spot" OR "blind spots" OR capabilities OR capability OR certainties OR certainty OR correct OR correctly OR difficulties OR difficulty OR erroneous OR error OR errors OR false OR faulty OR flawed OR imprecise* OR imprecision OR inaccurac* OR inaccurate* OR incorrect OR incorrectly OR judgment OR judgments OR mistake* OR precise* OR precision OR "strict criteria" OR "stringent criteria" OR uncertainties OR uncertainty OR wrong)) OR ((dispensing OR prescribe* OR prescribing OR prescription OR prescriptions OR prescriptive) AND (annual OR annually OR behavior* OR behaviour* OR improper OR improperly

OR over-consum* OR over-use* OR overconsum* OR overuse* OR pattern OR patterns OR proper OR properly OR trend OR trends OR under-consum* OR under-use* OR underconsum* OR underuse*))) AND TS=(amoxicillin* OR amoxicilline* OR amoxy* OR amoxycillin* OR amoxycilline* OR anti-bacteria* OR anti-biotic* OR anti-infect* OR anti-microbial* OR anti-mycobacteria* OR antibacteria* OR antibiotic* OR antiinfect* OR antimicrobial* OR antimycobacteria* OR azithromycin OR azitromicina OR azythromycin OR bacteriocid* OR "cef triaxone" OR cefatriaxone OR cefdinir OR cefotaxim OR cefotaxime OR cefotriaxone OR ceftriaxon OR ceftriaxone OR cephotaxim OR ciprofloxacin OR microbicide OR microbicides OR penicillin OR penicillins) AND PY=(2000-2023)

Cochrane CENTRAL Register of Controlled Trials (Wiley):

https://ckm.vumc.org/ckm/diglib/ckmres.html?rid=6610

Date Last Searched: 7/19/2023

Results: 398

Limits: Limited to children, Publication dates 2000-2023

([mh "Otitis Media"] OR [mh "Otitis Media, Suppurative"] OR ("infected middle ear" OR "infected middle ears" OR "infectious middle ear" OR "inflamed middle ear" OR "inflammation of middle ear" OR "inflammation of middle ears" OR "inflammation of the middle ear" OR "inflammation of the middle ears" OR "inflammatory middle ear" OR "inflammatory middle ears" OR "media otitis" OR (middle NEXT ear NEXT infect*) OR (middle NEXT ear NEXT inflam*) OR "middle otitis" OR (otitis NEXT media*) OR "otitis middle"):ti,ab,kw) AND ([mh "Antimicrobial Stewardship"] OR [mh "Decision Making, Shared"] OR [mh "Diagnostic Errors"] OR [mh "Drug Administration Schedule"] OR [mh "Duration of Therapy"] OR [mh "Guidelines as Topic"] OR [mh "Inappropriate Prescribing"] OR [mh "Observation"] OR [mh "Unnecessary Procedures"] OR [mh "Watchful Waiting"] OR ("1 course" OR "2 courses" OR "3 courses" OR "4 courses" OR "5 courses" OR (AAP NEXT recommend*) OR "active surveillance" OR "administration

schedule" OR "administration schedules" OR "American Academy of Pediatrics" OR "amoxicillin course" OR "antibiotic course" OR "antibiotic courses" OR "antibiotic days" OR "antibiotics longer" OR appropriate* OR "complete course" OR "complete courses" OR "complete drug" OR "complete drugs" OR "completed course" OR "completed drug" OR (completed NEXT oral*) OR (completed NEXT therap*) OR "completed treatment" OR "completing antibiotic" OR (completing NEXT therap*) OR "completing treatment" OR "course amoxycillin" OR "course antibacterial" OR "course antibiotic" OR "course antibiotics" OR "course antimicrobial" OR "course antimicrobials" OR "course daily" OR (course NEXT length*) OR "course of amoxicillin" OR "course of amoxycillin" OR "course of antibacterial" OR "course of antibiotic" OR "course of antibiotics" OR "course of antimicrobial" OR "course of antimicrobials" OR (course NEXT of NEXT oral*) OR "course of penicillin" OR (course NEXT of NEXT therap*) OR "course of treatment" OR (course NEXT oral*) OR "course regimen" OR "course regimens" OR "courses longer" OR "courses of antibiotic" OR "courses of antibiotics" OR "courses of antimicrobial" OR "courses of antimicrobials" OR (courses NEXT of NEXT oral*) OR (courses NEXT of NEXT therap*) OR "courses of treatment" OR "courses prescribed" OR "daily course" OR (daily NEXT oral*) OR "daily treatment" OR "day amoxicillin" OR "day amoxycillin" OR "day antibiotic" OR "day course" OR (day NEXT oral*) OR "day penicillin" OR "day regimen" OR "day regimens" OR (day NEXT therap*) OR "day treatment" OR "days course" OR "days of amoxicillin" OR "days of amoxycillin" OR "days of antibiotic" OR "days of antibiotics" OR (days NEXT of NEXT oral*) OR (days NEXT of NEXT therap*) OR "days of treatment" OR (days NEXT therap*) OR "days treatment" OR delay* OR "diagnoses criteria" OR "diagnosing ability" OR "diagnosing criteria" OR "diagnosis abilities" OR "diagnosis ability" OR (diagnosis NEXT behavior*) OR (diagnosis NEXT behaviour*) OR "diagnosis criteria" OR "diagnosis skill" OR "diagnosis skills" OR "diagnosis validity" OR "diagnostic abilities" OR "diagnostic ability" OR (diagnostic NEXT behavior*) OR (diagnostic NEXT behaviour*) OR "diagnostic criteria" OR "diagnostic criterion" OR "diagnostic skill" OR "diagnostic skills" OR "diagnostic validity" OR "diagnostical criteria" OR "diagnostical criterion" OR

"diagnostical validity" OR "difficult to diagnose" OR duration OR durations OR expectant OR "extended treatment" OR "extending treatment" OR (failed NEXT diagnos*) OR (failure NEXT to NEXT diagnos*) OR "first course" OR "first prescription" OR "five courses" OR "four courses" OR "fourth course" OR "full course" OR "full courses" OR "full treatment" OR "full treatments" OR guidance OR guideline OR guidelines OR immediacy OR immediate OR immediately OR (improper NEXT diagnos*) OR inappropriate* OR "initial amoxicillin" OR "initial antibacterial" OR "initial antibiotic" OR "initial antibiotics" OR "initial antimicrobial" OR "initial course" OR "initial drug" OR "initial penicillin" OR (initial NEXT therap*) OR "initial treatment" OR "initial treatments" OR judicious OR (length NEXT of NEXT therap*) OR "length of treatment" OR "long course" OR "long courses" OR "long drug" OR "long drugs" OR "long regimens" OR "long term amoxicillin" OR "long term antibiotic" OR "long term antibiotics" OR "long term penicillin" OR (long NEXT term NEXT therap*) OR "long term treatment" OR "long term treatments" OR "long treatment" OR "longer course" OR "longer courses" OR "longer regimens" OR "longer treatment" OR "longer treatments" OR mis-diagnos* OR misdiagnos* OR (missed NEXT diagnos*) OR "month course" OR "month treatment" OR "monthly courses" OR (months NEXT of NEXT oral*) OR (months NEXT of NEXT therap*) OR "months of treatment" OR "months treatment" OR "National Institute for Health and Care Excellence" OR "national recommendation" OR "national recommendations" OR (NICE NEXT recommend*) OR nonguideline OR "number of courses" OR "number of prescribed" OR "number of prescriptions" OR observation OR "one course" OR "one prescription" OR (optimal NEXT length*) OR "oral course" OR "oral regimen" OR "oral regimens" OR over-diagnos* OR over-prescrib* OR over-prescription OR over-prescriptions OR overdiagnos* OR overprescrib* OR overprescription OR (parent NEXT decision*) OR (parents NEXT decision*) OR (patient NEXT decision*) OR (patients NEXT decision*) OR "penicillin course" OR "penicillin courses" OR "prescribed daily" OR "prescribing habits" OR "prescribing practice" OR "prescribing practices" OR "prescribing rate" OR "prescribing rates" OR "prescription habits" OR (prescription NEXT length*) OR

"prescription practice" OR "prescription practices" OR "prescription rate" OR "prescription rates" OR "prescriptive practice" OR "prescriptive practices" OR "prolonged antibiotic" OR "prolonged course" OR "prolonged courses" OR "prolonged medication" OR "prolonged penicillin" OR (prolonged NEXT therap*) OR "prolonged treatment" OR (proper NEXT diagnos*) OR (properly NEXT diagnos*) OR prudent OR "rate of antibiotic" OR "rate of antibiotics" OR "rate of prescribing" OR "rate of prescription" OR "rates of antibiotic" OR "rates of antibiotics" OR "rates of prescribing" OR "regimens of antibiotic" OR "regimens of antibiotics" OR "safety net antibiotic" OR "second course" OR "second prescription" OR "second treatment" OR (shared NEXT decision*) OR "short antibiotic" OR "short antibiotics" OR "short course" OR "short courses" OR "short regimen" OR "short regimens" OR "short term antibiotic" OR "short term antibiotics" OR (short NEXT term NEXT oral*) OR "short term penicillin" OR "short term treatment" OR "short term treatments" OR "shortened antimicrobial" OR "shortened course" OR "shortened courses" OR (shortened NEXT therap*) OR "shortened treatment" OR "shorter antibiotic" OR "shorter course" OR "shorter courses" OR "shorter prescription" OR "shorter regimen" OR "shorter regimens" OR "shorter treatment" OR "standard courses" OR "standard regimen" OR "standard regimens" OR steward* OR "therapeutic recommendation" OR "therapeutic recommendations" OR "therapy courses" OR "therapy recommendation" OR "therapy regimens" OR "therapy recommendations" OR "three courses" OR "treatment course" OR "treatment courses" OR "treatment day" OR "treatment days" OR (treatment NEXT length*) OR "treatment recommendation" OR "treatment recommendations" OR "treatment regimen" OR "treatment regimens" OR "treatment time" OR "two courses" OR "two regimens" OR "two treatments" OR under-diagnos* OR under-prescrib* OR under-prescription OR under-prescriptions OR underdiagnos* OR underprescrib* OR underprescription OR unnecessary OR wait* OR watchful OR watching OR "week antibiotic" OR "week course" OR "weeks course" OR "weeks of antibiotic" OR withheld OR withhold*):ti,ab,kw OR (diagnos*:ti,ab,kw AND (accurac* OR accurate* OR "blind spot" OR "blind spots" OR capabilities OR capability OR certainties OR certainty OR correct OR correctly OR difficulties OR

difficulty OR erroneous OR error OR errors OR false OR faulty OR flawed OR imprecise* OR imprecision OR inaccurac* OR inaccurate* OR incorrect OR incorrectly OR judgment OR judgments OR mistake* OR precise* OR precision OR "strict criteria" OR "stringent criteria" OR uncertainties OR uncertainty OR wrong):ti,ab,kw) OR (([mh "Drug Prescriptions"] OR (dispensing OR prescribe* OR prescribing OR prescription OR prescriptions OR prescriptive):ti,ab,kw) AND ([mh "Practice Patterns, Physicians'"] OR (annual OR annually OR behavior* OR behaviour* OR improper OR improperly OR over-consum* OR over-use* OR overconsum* OR overuse* OR pattern OR patterns OR proper OR properly OR trend OR trends OR under-consum* OR under-use* OR underconsum* OR underuse*):ti,ab,kw))) AND ([mh "Amoxicillin"] OR [mh "Anti-Bacterial Agents"] OR [mh "Anti-Infective Agents"] OR [mh "Azithromycin"] OR [mh "Cefdinir"] OR [mh "Ceftriaxone"] OR [mh "Cefotaxime"] OR [mh "Ciprofloxacin"] OR [mh "Penicillins"] OR (amoxicillin* OR amoxicilline* OR amoxy* OR amoxycillin* OR amoxycilline* OR anti-bacteria* OR anti-biotic* OR anti-infect* OR anti-microbial* OR anti-mycobacteria* OR antibacteria* OR antibiotic* OR antiinfect* OR antimicrobial* OR antimycobacteria* OR azithromycin OR azitromicina OR azythromycin OR bacteriocid* OR "cef triaxone" OR cefatriaxone OR cefdinir OR cefotaxim OR cefotaxime OR cefotriaxone OR ceftriaxon OR ceftriaxone OR cephotaxim OR ciprofloxacin OR microbicide OR microbicides OR penicillin OR penicillins):ti,ab,kw) NOT ([mh "Adult"] NOT ([mh "Adolescent"] OR [mh "Child"] OR [mh "Infant"] OR [mh "Pediatrics"]))

Cochrane Database of Systematic Reviews (Wiley):

https://ckm.vumc.org/ckm/diglib/ckmres.html?rid=6610

Date Last Searched: 7/19/2023

Results: 23

Limits: Limited to children, Publication dates 1/1/2000-6/30/2023

([mh "Otitis Media"] OR [mh "Otitis Media, Suppurative"] OR ("infected middle ear" OR "infected middle ears" OR "infectious middle ear" OR "inflamed middle ear" OR "inflammation of middle ear" OR "inflammation of middle ears" OR "inflammation of the middle ear" OR "inflammation of the middle ears" OR "inflammatory middle ear" OR "inflammatory middle ears" OR "media otitis" OR (middle NEXT ear NEXT infect*) OR (middle NEXT ear NEXT inflam*) OR "middle otitis" OR (otitis NEXT media*) OR "otitis middle"):ti,ab,kw) AND ([mh "Antimicrobial Stewardship"] OR [mh "Decision Making, Shared"] OR [mh "Diagnostic Errors"] OR [mh "Drug Administration Schedule"] OR [mh "Duration of Therapy"] OR [mh "Guidelines as Topic"] OR [mh "Inappropriate Prescribing"] OR [mh "Observation"] OR [mh "Unnecessary Procedures"] OR [mh "Watchful Waiting"] OR ("1 course" OR "2 courses" OR "3 courses" OR "4 courses" OR "5 courses" OR (AAP NEXT recommend*) OR "active surveillance" OR "administration schedule" OR "administration schedules" OR "American Academy of Pediatrics" OR "amoxicillin course" OR "antibiotic course" OR "antibiotic courses" OR "antibiotic days" OR "antibiotics longer" OR appropriate* OR "complete course" OR "complete courses" OR "complete drug" OR "complete drugs" OR "completed course" OR "completed drug" OR (completed NEXT oral*) OR (completed NEXT therap*) OR "completed treatment" OR "completing antibiotic" OR (completing NEXT therap*) OR "completing treatment" OR "course amoxycillin" OR "course antibacterial" OR "course antibiotic" OR "course antibiotics" OR "course antimicrobial" OR "course antimicrobials" OR "course daily" OR (course NEXT length*) OR "course of amoxicillin" OR "course of amoxycillin" OR "course of antibacterial" OR "course of antibiotic" OR "course of antibiotics" OR "course of antimicrobial" OR "course of antimicrobials" OR (course NEXT of NEXT oral*) OR "course of penicillin" OR (course NEXT of NEXT therap*) OR "course of treatment" OR (course NEXT oral*) OR "course regimen" OR "course regimens" OR "courses longer" OR "courses of antibiotic" OR "courses of antibiotics" OR "courses of antimicrobial" OR "courses of antimicrobials" OR (courses NEXT of NEXT oral*) OR (courses NEXT of NEXT therap*) OR "courses of treatment" OR "courses prescribed" OR "daily course" OR (daily NEXT oral*) OR "daily treatment" OR

"day amoxicillin" OR "day amoxycillin" OR "day antibiotic" OR "day course" OR (day NEXT oral*) OR "day penicillin" OR "day regimen" OR "day regimens" OR (day NEXT therap*) OR "day treatment" OR "days course" OR "days of amoxicillin" OR "days of amoxycillin" OR "days of antibiotic" OR "days of antibiotics" OR (days NEXT of NEXT oral*) OR (days NEXT of NEXT therap*) OR "days of treatment" OR (days NEXT therap*) OR "days treatment" OR delay* OR "diagnoses criteria" OR "diagnosing ability" OR "diagnosing criteria" OR "diagnosis abilities" OR "diagnosis ability" OR (diagnosis NEXT behavior*) OR (diagnosis NEXT behaviour*) OR "diagnosis criteria" OR "diagnosis skill" OR "diagnosis skills" OR "diagnosis validity" OR "diagnostic abilities" OR "diagnostic ability" OR (diagnostic NEXT behavior*) OR (diagnostic NEXT behaviour*) OR "diagnostic criteria" OR "diagnostic criterion" OR "diagnostic skill" OR "diagnostic skills" OR "diagnostic validity" OR "diagnostical criteria" OR "diagnostical criterion" OR "diagnostical validity" OR "difficult to diagnose" OR duration OR durations OR expectant OR "extended treatment" OR "extending treatment" OR (failed NEXT diagnos*) OR (failure NEXT to NEXT diagnos*) OR "first course" OR "first prescription" OR "five courses" OR "four courses" OR "fourth course" OR "full course" OR "full courses" OR "full treatment" OR "full treatments" OR guidance OR guideline OR guidelines OR immediacy OR immediate OR immediately OR (improper NEXT diagnos*) OR inappropriate* OR "initial amoxicillin" OR "initial antibacterial" OR "initial antibiotic" OR "initial antibiotics" OR "initial antimicrobial" OR "initial course" OR "initial drug" OR "initial penicillin" OR (initial NEXT therap*) OR "initial treatment" OR "initial treatments" OR judicious OR (length NEXT of NEXT therap*) OR "length of treatment" OR "long course" OR "long courses" OR "long drug" OR "long drugs" OR "long regimens" OR "long term amoxicillin" OR "long term antibiotic" OR "long term antibiotics" OR "long term penicillin" OR (long NEXT term NEXT therap*) OR "long term treatment" OR "long term treatments" OR "long treatment" OR "longer course" OR "longer courses" OR "longer regimens" OR "longer treatment" OR "longer treatments" OR mis-diagnos* OR misdiagnos* OR (missed NEXT diagnos*) OR "month course" OR "month treatment" OR "monthly courses" OR (months NEXT of

NEXT oral*) OR (months NEXT of NEXT therap*) OR "months of treatment" OR "months treatment" OR "National Institute for Health and Care Excellence" OR "national recommendation" OR "national recommendations" OR (NICE NEXT recommend*) OR nonguideline OR "number of courses" OR "number of prescribed" OR "number of prescriptions" OR observation OR "one course" OR "one prescription" OR (optimal NEXT length*) OR "oral course" OR "oral regimen" OR "oral regimens" OR over-diagnos* OR over-prescrib* OR over-prescription OR over-prescriptions OR overdiagnos* OR overprescrib* OR overprescription OR (parent NEXT decision*) OR (parents NEXT decision*) OR (patient NEXT decision*) OR (patients NEXT decision*) OR "penicillin course" OR "penicillin courses" OR "prescribed daily" OR "prescribing habits" OR "prescribing practice" OR "prescribing practices" OR "prescribing rate" OR "prescribing rates" OR "prescription habits" OR (prescription NEXT length*) OR "prescription practice" OR "prescription practices" OR "prescription rate" OR "prescription rates" OR "prescriptive practice" OR "prescriptive practices" OR "prolonged antibiotic" OR "prolonged course" OR "prolonged courses" OR "prolonged medication" OR "prolonged penicillin" OR (prolonged NEXT therap*) OR "prolonged treatment" OR (proper NEXT diagnos*) OR (properly NEXT diagnos*) OR prudent OR "rate of antibiotic" OR "rate of antibiotics" OR "rate of prescribing" OR "rate of prescription" OR "rates of antibiotic" OR "rates of antibiotics" OR "rates of prescribing" OR "regimens of antibiotic" OR "regimens of antibiotics" OR "safety net antibiotic" OR "second course" OR "second prescription" OR "second treatment" OR (shared NEXT decision*) OR "short antibiotic" OR "short antibiotics" OR "short course" OR "short courses" OR "short regimen" OR "short regimens" OR "short term antibiotic" OR "short term antibiotics" OR (short NEXT term NEXT oral*) OR "short term penicillin" OR "short term treatment" OR "short term treatments" OR "shortened antimicrobial" OR "shortened course" OR "shortened courses" OR (shortened NEXT therap*) OR "shortened treatment" OR "shorter antibiotic" OR "shorter course" OR "shorter courses" OR "shorter prescription" OR "shorter regimen" OR "shorter regimens" OR "shorter treatment" OR "standard courses" OR "standard regimen" OR "standard regimens" OR steward* OR

"therapeutic recommendation" OR "therapeutic recommendations" OR "therapy courses" OR "therapy recommendation" OR "therapy regimens" OR "therapy recommendations" OR "three courses" OR "treatment course" OR "treatment courses" OR "treatment day" OR "treatment days" OR (treatment NEXT length*) OR "treatment recommendation" OR "treatment recommendations" OR "treatment regimen" OR "treatment regimens" OR "treatment time" OR "two courses" OR "two regimens" OR "two treatments" OR under-diagnos* OR under-prescrib* OR under-prescription OR under-prescriptions OR underdiagnos* OR underprescrib* OR underprescription OR unnecessary OR wait* OR watchful OR watching OR "week antibiotic" OR "week course" OR "weeks course" OR "weeks of antibiotic" OR withheld OR withhold*):ti,ab,kw OR (diagnos*:ti,ab,kw AND (accurac* OR accurate* OR "blind spot" OR "blind spots" OR capabilities OR capability OR certainties OR certainty OR correct OR correctly OR difficulties OR difficulty OR erroneous OR error OR errors OR false OR faulty OR flawed OR imprecise* OR imprecision OR inaccurac* OR inaccurate* OR incorrect OR incorrectly OR judgment OR judgments OR mistake* OR precise* OR precision OR "strict criteria" OR "stringent criteria" OR uncertainties OR uncertainty OR wrong):ti,ab,kw) OR (([mh "Drug Prescriptions"] OR (dispensing OR prescribe* OR prescribing OR prescription OR prescriptions OR prescriptive):ti,ab,kw) AND ([mh "Practice Patterns, Physicians'"] OR (annual OR annually OR behavior* OR behaviour* OR improper OR improperly OR over-consum* OR over-use* OR overconsum* OR overuse* OR pattern OR patterns OR proper OR properly OR trend OR trends OR under-consum* OR under-use* OR underconsum* OR underuse*):ti,ab,kw))) AND ([mh "Amoxicillin"] OR [mh "Anti-Bacterial Agents"] OR [mh "Anti-Infective Agents"] OR [mh "Azithromycin"] OR [mh "Cefdinir"] OR [mh "Ceftriaxone"] OR [mh "Cefotaxime"] OR [mh "Ciprofloxacin"] OR [mh "Penicillins"] OR (amoxicillin* OR amoxicilline* OR amoxy* OR amoxycillin* OR amoxycilline* OR anti-bacteria* OR anti-biotic* OR anti-infect* OR anti-microbial* OR anti-mycobacteria* OR antibacteria* OR antibiotic* OR antiinfect* OR antimicrobial* OR antimycobacteria* OR azithromycin OR azitromicina OR azythromycin OR bacteriocid* OR "cef triaxone" OR cefatriaxone OR cefdinir OR cefotaxim OR

cefotaxime OR cefotriaxone OR ceftriaxon OR ceftriaxone OR cephotaxim OR ciprofloxacin OR microbicide OR microbicides OR penicillin OR penicillins):ti,ab,kw) NOT ([mh "Adult"] NOT ([mh "Adolescent"] OR [mh "Child"] OR [mh "Infant"] OR [mh "Pediatrics"]))

Table S1. All included meta-analysis studies.

Publication Author & Year	Study Type	Total # of Subjects	Data Obtained
Abuali 2019	Cross Sectional	59	Watchful Waiting (Proportions)
Alzahrani 2018	Cross Sectional	5,200,000	Annual Encounters
Anderson 2023	RCT[a]	101	Watchful Waiting (Effectiveness)
Barrera 2019	Case Series	365	Severity of Infection (Proportion), Watchful Waiting (Proportions)
Barrera 2019	Case Series	246	Bilateral Infection (Proportions)
Boatright 2015	Retrospective	100	Watchful Waiting (Proportions)
Bondy 2000	Cohort Study	24,677	Subject Age (Proportions)
Bradley 2021	Cohort Study	862	Watchful Waiting (Effectiveness)
Bradley 2021	Cohort Study	1,053	Subject Age (Proportions)
Brinker 2019	Retrospective	520	Diagnostic Accuracy (Proportions)
Brinker 2019	Retrospective	448	Watchful Waiting (Proportions)
Brinker 2019	Retrospective	436	Severity of Infection (Proportion)
Chao 2008	RCT	206	Bilateral Infection (Proportions), Severity of Infection (Proportion)
Chiappini 2020	Survey	1,079	Watchful Waiting (Proportions)
Coco 2009	Case Series	8,325	Watchful Waiting (Proportions)
Coco 2010	Cohort Study	1,114	Watchful Waiting (Proportions), Watchful Waiting (Effectiveness)
Cox 2008	Case Series	66	Diagnostic Accuracy (Proportions)
Crowson 2023	Cohort Study	639	Diagnostic Accuracy (Proportions)
Cushen 2020	Cohort Study	286,574	Subject Age (Proportions)
Daggett 2022	QI[b]	9,026	Duration of Tx Reduction (Effectiveness)
Damoiseaux 2000	RCT	240	Bilateral Infection (Proportions)
Di 2017	Cohort Study	247	Watchful Waiting (Proportions)
Dona 2018	Experimental	573	Watchful Waiting (Proportions), Watchful Waiting (Effectiveness)
Esposito 2007	Cohort Study	1,555	Subject Age (Proportions)
Fiks 2015	Cohort Study	41,391	Subject Age (Proportions)
Fischer 2009	Case Series	144	Bilateral Infection (Proportions), Delayed Prescription (Proportions)
Fleming-Dutra 2014	Cohort Study	19,200,000	Subject Age (Proportions), Annual Encounters
Frost 2020	Case Series	926	Watchful Waiting (Proportions)
Frost 2021	Survey	84	Delayed Prescription (Proportions)
Frost 2022	Cohort Study	388	Duration of Tx (Proportions)
Frost 2022	Cohort Study	503	Duration of Tx Reduction (Effectiveness)
Frost 2022	Experimental	1,051,007	Delayed Prescription (Proportions), Subject Age (Proportions)
Gaboury 2010	RCT	488	Severity of Infection (Proportion)
Garbutt 2003	Cohort Study	573	Diagnostic Accuracy (Proportions), Watchful Waiting (Proportions)

GarciaVentura 2022	Case Series	1,036	Delayed Prescription (Proportions)
Groth 2011	Case Series	577	Severity of Infection (Proportion)
Gurnaney 2004	Survey	87	Severity of Infection (Proportion)
Hoberman 2011	RCT	291	Bilateral Infection (Proportions), Severity of Infection (Proportion)
Hoberman 2016	RCT	515	Bilateral Infection (Proportions), Severity of Infection (Proportion)
Hu 2022	Cohort Study	15,330,024	Annual Encounters
Hullegie 2021	Cohort Study	6,781	Subject Age (Proportions)
Islam 2020	Case Series	120	Watchful Waiting (Proportions)
Jokinen 2023	Survey	888	Observation (Proportions)
Kalu 2011	Cohort Study	420	Watchful Waiting (Proportions)
Kalu 2011	Cohort Study	294	Bilateral Infection (Proportions), Severity of Infection (Proportion)
Kautz-Freimuth 2015	Survey	104	Observation (Proportions)
Kautz-Freimuth 2015	Survey	278	Subject Age (Proportions)
King 2021	Cohort Study	6,252,288	Annual Encounters
Kleinman 2021	RCT	197	Diagnostic Accuracy (Proportions)
Kleinman 2021	RCT	375	Diagnostic Accuracy (Effectiveness)
Kuruvilla 2013	Cohort Study	362	Diagnostic Accuracy (Effectiveness)
Kuruvilla 2013	Cohort Study	826	Diagnostic Accuracy (Proportions)
LeSaux 2005	RCT	512	Watchful Waiting (Proportions)
Linsk 2004	Survey	125	Watchful Waiting (Proportions)
Little 2001	RCT	315	Watchful Waiting (Effectiveness)
Marchetti 2005	Cohort Study	1,099	Watchful Waiting (Proportions)
Marom 2014	Cohort Study	6,209,019	Subject Age (Proportions)
McCormick 2005	RCT	223	Severity of Infection (Proportion), Watchful Waiting (Proportions)
McGrath 2013	Cohort Study	4,629,460	Watchful Waiting (Proportions)
McGrath 2023	Cohort Study	1,291,663	Subject Age (Proportions)
McGrath 2023	Cohort Study	1,291,663	Duration of Tx (Proportions)
Merenstein 2005	Cross Sectional	328	Watchful Waiting (Effectiveness)
Nedved 2023	QI	403	Watchful Waiting (Proportions)
Nedved 2023	Cohort Study	14,728	Duration of Tx Reduction (Effectiveness)
Neumark 2007	RCT	179	Observation (Proportions), Severity of Infection (Proportion)
Oliveira 2021	Retrospective	1,646	Bilateral Infection (Proportions), Watchful Waiting (Proportions)
Olsen 2020	Cross Sectional	278	Severity of Infection (Proportion), Watchful Waiting (Proportions)
Palma 2015	Case Series	4,573	Subject Age (Proportions)
Pichichero 2001	Cohort Study	524	Diagnostic Accuracy (Proportions)
Pichichero 2002	Experimental	2,190	Diagnostic Accuracy (Proportions)
Rosenfeld 2002	Survey	135	Diagnostic Accuracy (Proportions), Severity of Infection (Proportion), Watchful Waiting (Proportions)

Rothman 2017	Case Series	1,449	Watchful Waiting (Proportions)
Rothman 2017	Case Series	1,493	Severity of Infection (Proportion)
Rothman 2017	Case Series	1,448	Bilateral Infection (Proportions)
Rothman 2017	Case Series	1,134	Subject Age (Proportions)
Ryborg 2013	Cohort Study	441	Severity of Infection (Proportion)
Ryborg 2013	Cohort Study	940	Subject Age (Proportions)
Shah-Becker 2018	Survey	76	Watchful Waiting (Proportions)
Shaikh 2011	Cohort Study	738	Diagnostic Accuracy (Proportions)
Shireman 2002	Cohort Study	9,177	Watchful Waiting (Proportions)
Siegel 2003	Experimental	175	Subject Age (Proportions), Watchful Waiting (Proportions)
Siegel 2003	Experimental	161	Delayed Prescription (Proportions)
Siegel 2006	Cohort Study	175	Watchful Waiting (Proportions)
Siegel 2006	Cohort Study	117	Delayed Prescription (Proportions)
Småbrekke 2002	Experimental	819	Subject Age (Proportions)
Smolinski 2022	Cohort Study	2,176,617	Watchful Waiting (Proportions)
Smolinski 2022	Cohort Study	482,927	Delayed Prescription (Proportions)
Spiro 2004	RCT	698	Watchful Waiting (Proportions)
Spiro 2006	RCT	132	Delayed Prescription (Proportions)
Spiro 2006	RCT	265	Watchful Waiting (Effectiveness)
Spiro 2006	RCT	283	Severity of Infection (Proportion)
Stevanovic 2010	RCT	314	Watchful Waiting (Proportions)
TÃ¤htinen 2011	RCT	319	Severity of Infection (Proportion), Watchful Waiting (Proportions)
Tähtinen 2011	RCT	315	Bilateral Infection (Proportions)
Tähtinen 2012	RCT	160	Observation (Proportions)
Talathi 2017	Cohort Study	103	Severity of Infection (Proportion), Subject Age (Proportions), Watchful Waiting (Proportions)
Thompson 2008	Retrospective	1,210,237	Subject Age (Proportions)
Tong 2018	Retrospective	17,641,449	Subject Age (Proportions)
Uhl 2021	QI	17,998	Duration of Tx Reduction (Effectiveness), Subject Age (Proportions)
Vernacchio 2006	Survey	276	Watchful Waiting (Proportions)
Vernacchio 2007	Survey	299	Watchful Waiting (Proportions)
Wolf 2022	QI	126	Watchful Waiting (Proportions)
Wolf 2022	QI	1,387	Watchful Waiting (Effectiveness)
Wyly 2023	Retrospective	271	Watchful Waiting (Proportions)

[a]RCT: Randomized Controlled Trial
[b]QI: Quality Improvement

Table S2. Overall total days of therapy saved from following AAP and NICE guidelines.

Guideline	Average Estimated Days of therapy	Days of therapy minimum	Days of therapy maximum
Current Practice	107,415,830	48,589,320	179,406,720
AAP[a] Recommendation	46,845,310	20,906,529	79,289,431
Days of therapy saved	60,570,520	27,682,791	100,114,289
% Decrease in days of therapy	56.4%	57.0%	55.8%
NICE[b] Guidelines	30,716,910	11,578,940	59,854,213
Days of therapy saved	76,698,920	37,010,380	
% Decrease in days of therapy	71.4%	76.2%	66.6%

[a]AAP: American Academy of Pediatrics

[b]NICE: National Institute for Health and Care Excellence

Figure S1. Preferred Reporting Items for Systematic Reviews and Meta-Analyses (PRISMA) Checklist

Section and Topic	Item #	Checklist item	Location where item is reported
**TITLE**			
Title	1	Identify the report as a systematic review.	Title Page
**ABSTRACT**			
Abstract	2	See the PRISMA 2020 for Abstracts checklist.	Abstracts Checklist
**INTRODUCTION**			
Rationale	3	Describe the rationale for the review in the context of existing knowledge.	Pg. 2
Objectives	4	Provide an explicit statement of the objective(s) or question(s) the review addresses.	Pg. 2
**METHODS**			
Eligibility criteria	5	Specify the inclusion and exclusion criteria for the review and how studies were grouped for the syntheses.	Pg. 2-3
Information sources	6	Specify all databases, registers, websites, organisations, reference lists and other sources searched or consulted to identify studies. Specify the date when each source was last searched or consulted.	Pg. 2
Search strategy	7	Present the full search strategies for all databases, registers and websites, including any filters and limits used.	Pg. 3 and Supplement
Selection process	8	Specify the methods used to decide whether a study met the inclusion criteria of the review, including how many reviewers screened each record and each report retrieved, whether they worked independently, and if applicable, details of automation tools used in the process.	Pg. 3
Data collection process	9	Specify the methods used to collect data from reports, including how many reviewers collected data from each report, whether they worked independently, any processes for obtaining or confirming data from study investigators, and if applicable, details of automation tools used in the process.	Pg. 3
Data items	10a	List and define all outcomes for which data were sought. Specify whether all results that were compatible with each outcome domain in each study were sought (e.g. for all measures, time points, analyses), and if not, the methods used to decide which results to collect.	Pg. 3-4
	10b	List and define all other variables for which data were sought (e.g. participant and intervention characteristics, funding sources). Describe any assumptions made about any missing or unclear information.	Pg. 4
Study risk of bias assessment	11	Specify the methods used to assess risk of bias in the included studies, including details of the tool(s) used, how many reviewers assessed each study and whether they worked independently, and if applicable, details of automation tools used in the process.	Pg. 3
Effect measures	12	Specify for each outcome the effect measure(s) (e.g. risk ratio, mean difference) used in the synthesis or presentation of results.	Pg. 4
Synthesis methods	13a	Describe the processes used to decide which studies were eligible for each synthesis (e.g. tabulating the study intervention characteristics and comparing against the planned groups for each synthesis (item #5)).	Pg .4
	13b	Describe any methods required to prepare the data for presentation or synthesis, such as handling of missing summary statistics, or data conversions.	Pg. 4
	13c	Describe any methods used to tabulate or visually display results of individual studies and syntheses.	Pg. 4

Section and Topic	Item #	Checklist item	Location where item is reported
	13d	Describe any methods used to synthesize results and provide a rationale for the choice(s). If meta-analysis was performed, describe the model(s), method(s) to identify the presence and extent of statistical heterogeneity, and software package(s) used.	Pg. 4
	13e	Describe any methods used to explore possible causes of heterogeneity among study results (e.g. subgroup analysis, meta-regression).	Pg. 5-6
	13f	Describe any sensitivity analyses conducted to assess robustness of the synthesized results.	Pg. 5-6
Reporting bias assessment	14	Describe any methods used to assess risk of bias due to missing results in a synthesis (arising from reporting biases).	Pg. 3
Certainty assessment	15	Describe any methods used to assess certainty (or confidence) in the body of evidence for an outcome.	Pg. 4
**RESULTS**			
Study selection	16a	Describe the results of the search and selection process, from the number of records identified in the search to the number of studies included in the review, ideally using a flow diagram.	Pg. 4-5, Supplement
	16b	Cite studies that might appear to meet the inclusion criteria, but which were excluded, and explain why they were excluded.	Supplement
Study characteristics	17	Cite each included study and present its characteristics.	Pg. 10-15, Supplement
Risk of bias in studies	18	Present assessments of risk of bias for each included study.	N/A
Results of individual studies	19	For all outcomes, present, for each study: (a) summary statistics for each group (where appropriate) and (b) an effect estimate and its precision (e.g. confidence/credible interval), ideally using structured tables or plots.	Pg. 5-9
Results of syntheses	20a	For each synthesis, briefly summarise the characteristics and risk of bias among contributing studies.	N/A
	20b	Present results of all statistical syntheses conducted. If meta-analysis was done, present for each the summary estimate and its precision (e.g. confidence/credible interval) and measures of statistical heterogeneity. If comparing groups, describe the direction of the effect.	Pg. 5
	20c	Present results of all investigations of possible causes of heterogeneity among study results.	Pg. 5
	20d	Present results of all sensitivity analyses conducted to assess the robustness of the synthesized results.	Pg. 5
Reporting biases	21	Present assessments of risk of bias due to missing results (arising from reporting biases) for each synthesis assessed.	N/A
Certainty of evidence	22	Present assessments of certainty (or confidence) in the body of evidence for each outcome assessed.	Pg. 6
**DISCUSSION**			
Discussion	23a	Provide a general interpretation of the results in the context of other evidence.	Pg. 8
	23b	Discuss any limitations of the evidence included in the review.	Pg. 9
	23c	Discuss any limitations of the review processes used.	Pg. 9
	23d	Discuss implications of the results for practice, policy, and future research.	Pg. 8, 10

Section and Topic	Item #	Checklist item	Location where item is reported
**OTHER INFORMATION**			
Registration and protocol	24a	Provide registration information for the review, including register name and registration number, or state that the review was not registered.	Pg. 11
	24b	Indicate where the review protocol can be accessed, or state that a protocol was not prepared.	Pg. 11
	24c	Describe and explain any amendments to information provided at registration or in the protocol.	N/A
Support	25	Describe sources of financial or non-financial support for the review, and the role of the funders or sponsors in the review.	Pg. 11
Competing interests	26	Declare any competing interests of review authors.	Pg. 11
Availability of data, code and other materials	27	Report which of the following are publicly available and where they can be found: template data collection forms; data extracted from included studies; data used for all analyses; analytic code; any other materials used in the review.	Pg. 11

Figure S2. Preferred Reporting Items for Systematic Reviews and Meta-Analyses (PRISMA) Abstracts Checklist

Section and Topic	Item #	Checklist item	Reported (Yes/No)
**TITLE**			
Title	1	Identify the report as a systematic review.	Yes
**BACKGROUND**			
Objectives	2	Provide an explicit statement of the main objective(s) or question(s) the review addresses.	Yes
**METHODS**			
Eligibility criteria	3	Specify the inclusion and exclusion criteria for the review.	Yes
Information sources	4	Specify the information sources (e.g. databases, registers) used to identify studies and the date when each was last searched.	Yes
Risk of bias	5	Specify the methods used to assess risk of bias in the included studies.	No
Synthesis of results	6	Specify the methods used to present and synthesise results.	Yes
**RESULTS**			
Included studies	7	Give the total number of included studies and participants and summarise relevant characteristics of studies.	Yes
Synthesis of results	8	Present results for main outcomes, preferably indicating the number of included studies and participants for each. If meta-analysis was done, report the summary estimate and confidence/credible interval. If comparing groups, indicate the direction of the effect (i.e. which group is favoured).	Yes
**DISCUSSION**			
Limitations of evidence	9	Provide a brief summary of the limitations of the evidence included in the review (e.g. study risk of bias, inconsistency and imprecision).	No
Interpretation	10	Provide a general interpretation of the results and important implications.	Yes
**OTHER**			
Funding	11	Specify the primary source of funding for the review.	N/A
Registration	12	Provide the register name and registration number.	Yes
